# *Chlamydia pneumoniae* and chronic asthma: Updated systematic review and meta-analysis of population attributable risk

**DOI:** 10.1371/journal.pone.0250034

**Published:** 2021-04-19

**Authors:** David L. Hahn

**Affiliations:** St Marys Hospital, Madison, Wisconsin, United States of America; Midwestern University, UNITED STATES

## Abstract

**Background:**

*Chlamydia pneumoniae* (Cp) is an obligate intracellular human respiratory pathogen producing persisting lung infection with a plausible link to asthma pathogenesis. The population attributable risk of potentially treatable Cp infection in asthma has not been reported.

**Methods:**

The author searched from 2000 to 2020 inclusive for previously un-reviewed and new cross sectional and prospective controlled studies of Cp biomarkers and chronic asthma in both children and adults. Qualitative descriptive results and quantitative estimates of population attributable risk for selected biomarkers (specific IgG, IgA and IgE) are presented.

**Findings:**

No large, long-term prospective population-based studies of Cp infection and asthma were identified. About half of case-control studies reported one or more significant associations of Cp biomarkers and chronic asthma. Heterogeneity of results by age group (pediatric v adult asthma), severity category (severe/uncontrolled, moderate/partly controlled, mild/controlled) and antibody isotype (specific IgG, IgA, IgE) were suggested by the qualitative results and confirmed by meta-analyses. The population attributable risks for Cp-specific IgG and IgA were nul in children and were 6% (95% confidence interval 2%-10%, p = 0.002) and 13% (9%-18%, p<0.00001) respectively in adults. In contrast to the nul or small population attributable risks for Cp-specific IgG and IgA, the population attributable risk for *C*. *pneumoniae*-specific IgE (children and adults combined) was 47% (39%-55%, p<0.00001). In the subset of studies that reported on asthma severity categories, Cp biomarkers were positively and significantly (P<0.00001) associated with asthma severity.

**Interpretation:**

*C*. *pneumoniae*-specific IgE is strongly associated with asthma and asthma severity, suggesting a possible mechanism linking chronic Cp infection with asthma in a subset of individuals with asthma. Infection biomarkers should be included in future macrolide treatment trials for severe and uncontrolled asthma.

## Introduction

Macrolides are now included in the Global Initiative for Asthma (GINA) [1], European Respiratory Society/American Thoracic Society (ERS/ATS) [2], and British Thoracic Society (BTS) [3] guidelines as treatment options for severe asthma. Macrolide mechanism(s) of action in asthma are currently unknown and could include immunomodulatory, anti-viral, or anti-microbial effects [4]. Information regarding the potential quantitative contribution of candidate organisms to asthma pathogenesis may aid in prioritizing research efforts into macrolide mechanisms of action and possibly help guide clinician prescribing of macrolides for severe asthma. Among the candidate target organisms is *Chlamydia pneumoniae* (Cp). Cp is an obligate intracellular human pathogen that was first described as a new species causing acute human respiratory infections (primarily bronchitis and pneumonia) in 1986 [5]. The first report associating possible Cp persistent infection or re-infection with asthma was published in 1991 [6]. Two subsequent reviews on associations of Cp and asthma, published in 1999 [7] and 2005 [8], reported on a growing body of evidence linking Cp biomarkers and chronic airway obstructive illnesses (asthma, chronic bronchitis and chronic obstructive pulmonary disease–COPD). Both reviews reported positive associations of infection biomarkers with asthma and highlighted the urgent need for further research to inform treatment guidelines. Neither of the reviews attempted to quantify the potential contribution of infection to asthma population burden. The purpose of the current systematic review and meta-analysis is to (1) tabulate observational studies of Cp biomarkers and chronic asthma in both children and adults that were published during and after 2005—the date of the most recent review [8]–and (2) calculate estimates for the population attributable risk (PAR) of selected Cp biomarkers in chronic asthma.

## Methods

The overall research question is What proportion of chronic stable asthma is potentially attributable to or influenced by chronic *Chlamydia pneumoniae* infection? Or In individuals diagnosed with chronic stable asthma, compared to non-asthma controls, do selected Cp infection biomarkers demonstrate an increased prevalence? Because direct microbiologic organism detection of deep lung infection is currently not feasible in population studies, this updated systematic review and meta-analysis focused on peripheral blood serologic biomarkers as potential surrogates for infection.

### Search strategy and selection criteria

For the updated systematic review, the author searched Pub Med, Scopus, CINAHL and The Cochrane Library using the search term “asthma and (*Chlamydophila pneumoniae* or *Chlamydia pneumoniae*)” limited to the years 2000 to October 2020. (see supplementary file S1 Appendix. Search algorithms for updated systematic review). The years 2000 to 2005 were included in case some studies published in those years had been overlooked by the 2005 review. The search returned 332 unique articles that were screened for inclusion as either case-control or prospective cohort studies of chronic asthma. To avoid confounding by acute infections in acute exacerbations, studies that reported only patients in exacerbation were excluded. Additionally, studies containing a mix of exacerbated and stable asthma patients, that did not report on chronic stable asthma separately, were excluded. Foreign language publications were included if they contained an English language abstract and sufficient biomarker data on cases and controls. A study was included in this updated review if it (1) reported on one or more biomarkers of Cp infection, (2) studied a population of subjects with clinician diagnosed chronic stable asthma, (3) included a non-asthmatic control group and (4) presented sufficient information to calculate biomarker prevalence in the chronic stable asthma case group and control groups separately. An additional criterion for inclusion as a prospective study was a follow up duration of at least five years. The author did not re-tabulate studies that had been reviewed in detail earlier (i.e., were contained in Table I of reference number 8).

**Fig 1:**
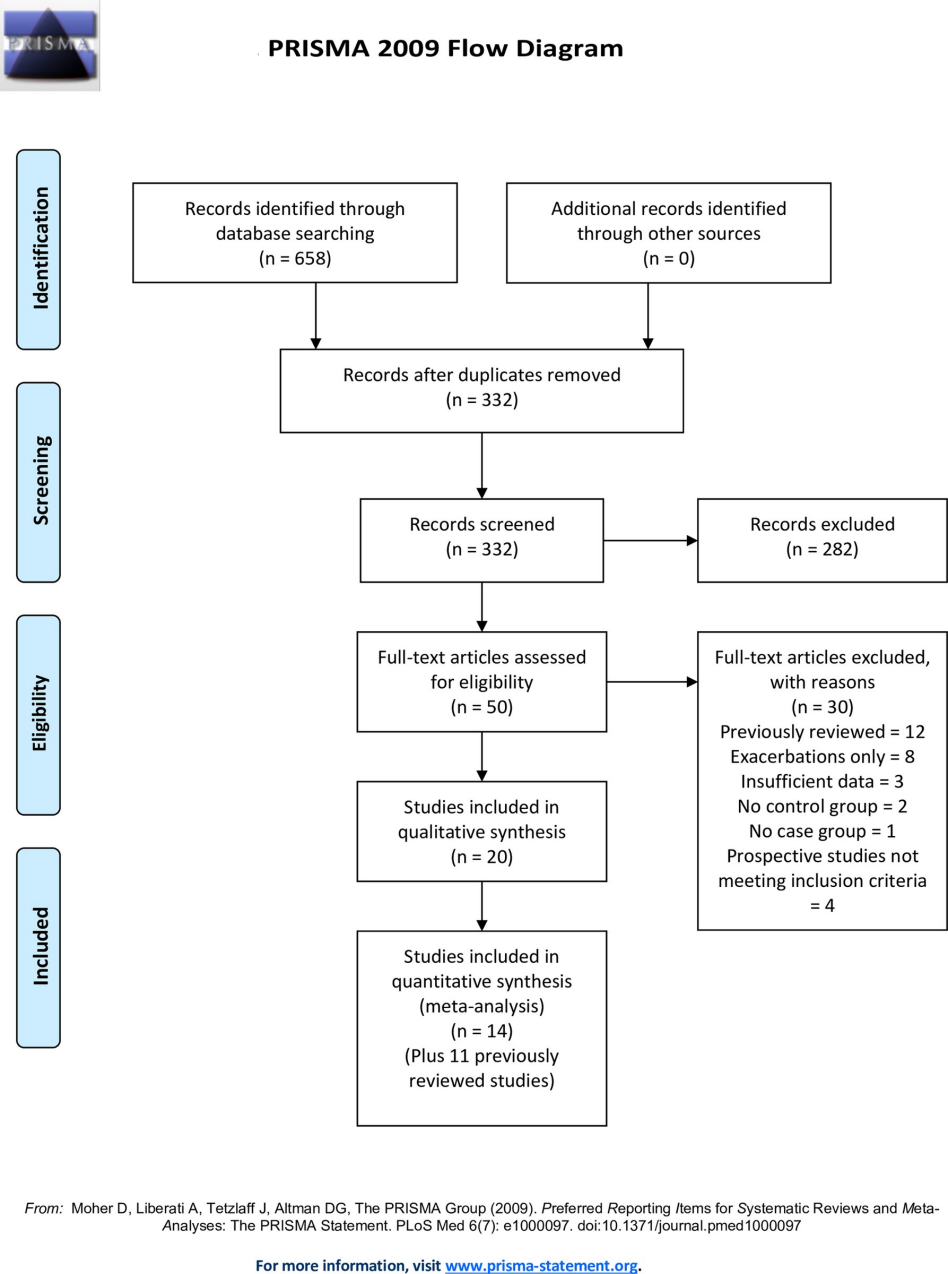
Pub Med search results (asthma and (*Chlamydia pneumoniae or Chlamydophila pneumoniae*)) limits 1/1/2000 to 10/2020.

Using these criteria 20 publications were included in this updated systematic review (Fig 1). Cross sectional studies were abstracted for study location (country), case and control definitions, microbiological method(s), main finding(s) and additional comments. Prospective studies were abstracted for location, study design, population studied, microbiological method(s), outcome definition(s), main finding(s) and additional comments. Microbiologic tests included culture, polymerase chain reaction (PCR) testing and/or various serologic tests. Selected methodologic issues for case-control studies are discussed in the text and presented systematically in supplementary file S2 Appendix for the studies included in the meta-analysis. This study did not involve human subjects and human subjects committee approval was not sought.

### Data analysis

Meta-analyses of population attributable risks (PAR) were estimated using Review Manager (RevMan) {Computer program} Version 5.4, The Cochrane Collaboration, 2020. Individual meta-analyses for Cp-specific IgG, IgA and IgE are reported. Appropriate data for meta-analysis were available from a total of 25 studies: 14 tabulated in this updated review and 11 tabulated in the 2005 review [8] (Fig 1). PAR was defined as the absolute difference between prevalence of biomarker positivity in the asthma case group and the non-asthma control group. For example, if 50% of asthma cases and 40% of controls are biomarker positive, the difference (50% minus 40% = 10%) is the population attributable risk. The PAR is an estimate of what percent of an outcome could possibly be prevented if a risk factor were to be removed from a population [9]. The risk difference between cases and controls was analyzed using the Mantel-Haenzel Fixed Effect method. Heterogeneity was assessed using the heterogeneity Chi-square test and the I^2^ statistic. Results are presented with 95% confidence intervals. Cp-specific IgG, IgA and IgE on peripheral blood were chosen for meta-analysis because they were by far the most commonly reported biomarkers. Peripheral blood is also the most accessible and reliable source for biomarker data in clinical practice and research. Cp IgM was not analyzed because it is primarily a marker for acute, not chronic, infection. Cp IgG and IgA were analyzed individually for child- and adult asthma separately. Due to the limited number of studies, Cp IgE was analyzed for both age groups combined. A subset of studies reported on asthma severity. Due to the limited number of such studies, a meta-analysis combining biomarkers and asthma age groups was performed to estimate and compare the PARs for severity subgroups. Study definitions for severity varied, e.g., some studies used a mild-moderate-severe nosology and others used a controlled-partly controlled-uncontrolled nosology. Therefore, severity was concatenated into mild/controlled, moderate/partly controlled and severe/uncontrolled categories. Biomarker classification (positive or negative), asthma case definitions and severity categories were recorded based on the criteria of the publication.

### Quality assessment of individual studies

**Fig 2:**
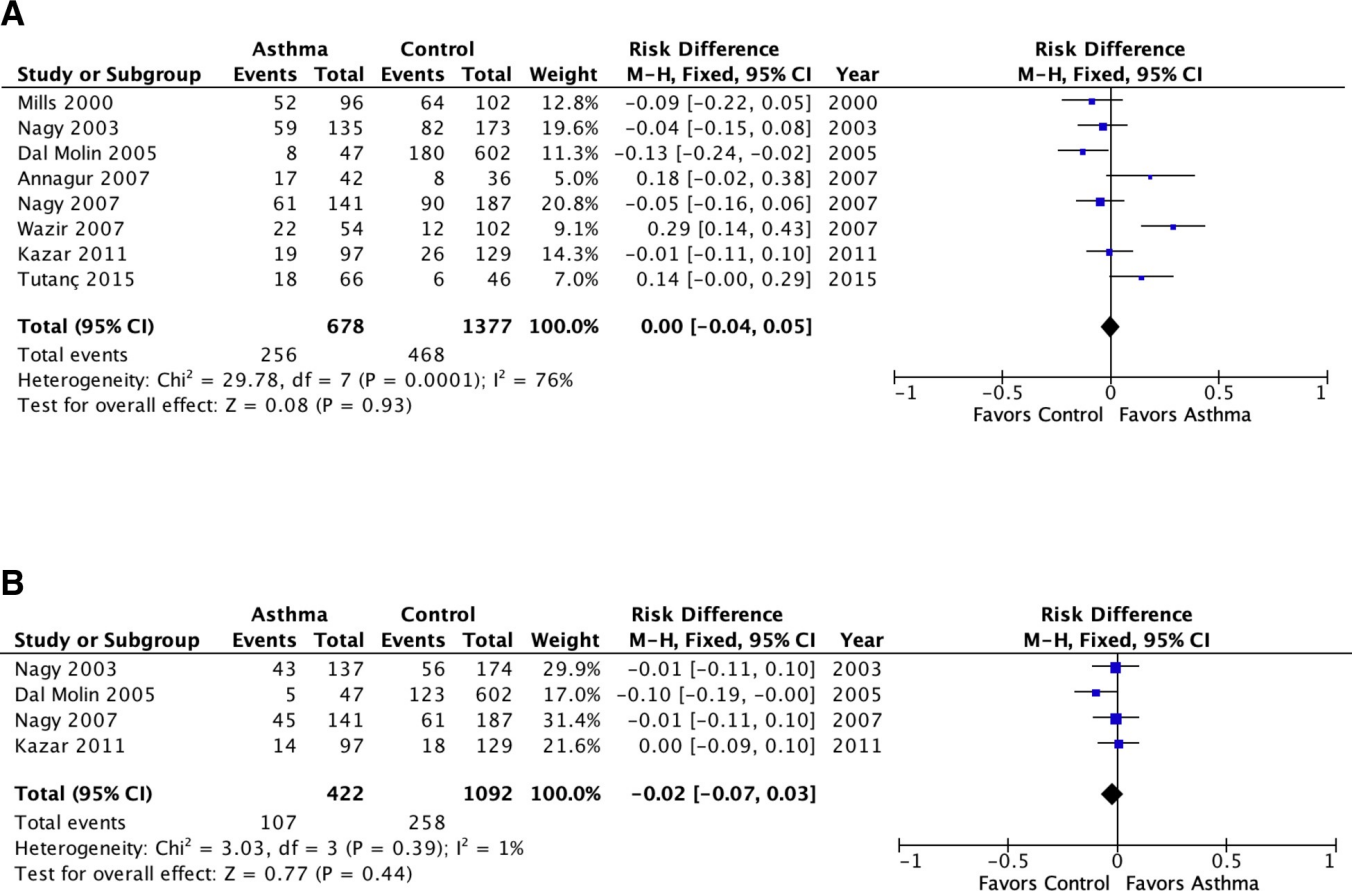
Meta-analysis of population attributable risk (*C*. *pneumoniae* biomarkers and asthma) in pediatric asthma. A: *C*. *pneumoniae-specific* IgG. B: *C*. *pneumoniae*-specific IgA. Population attributable risk varies from 0 (0%: contributes to none of the disease) to 1 (100%: contributes to all of the disease).

**Fig 3:**
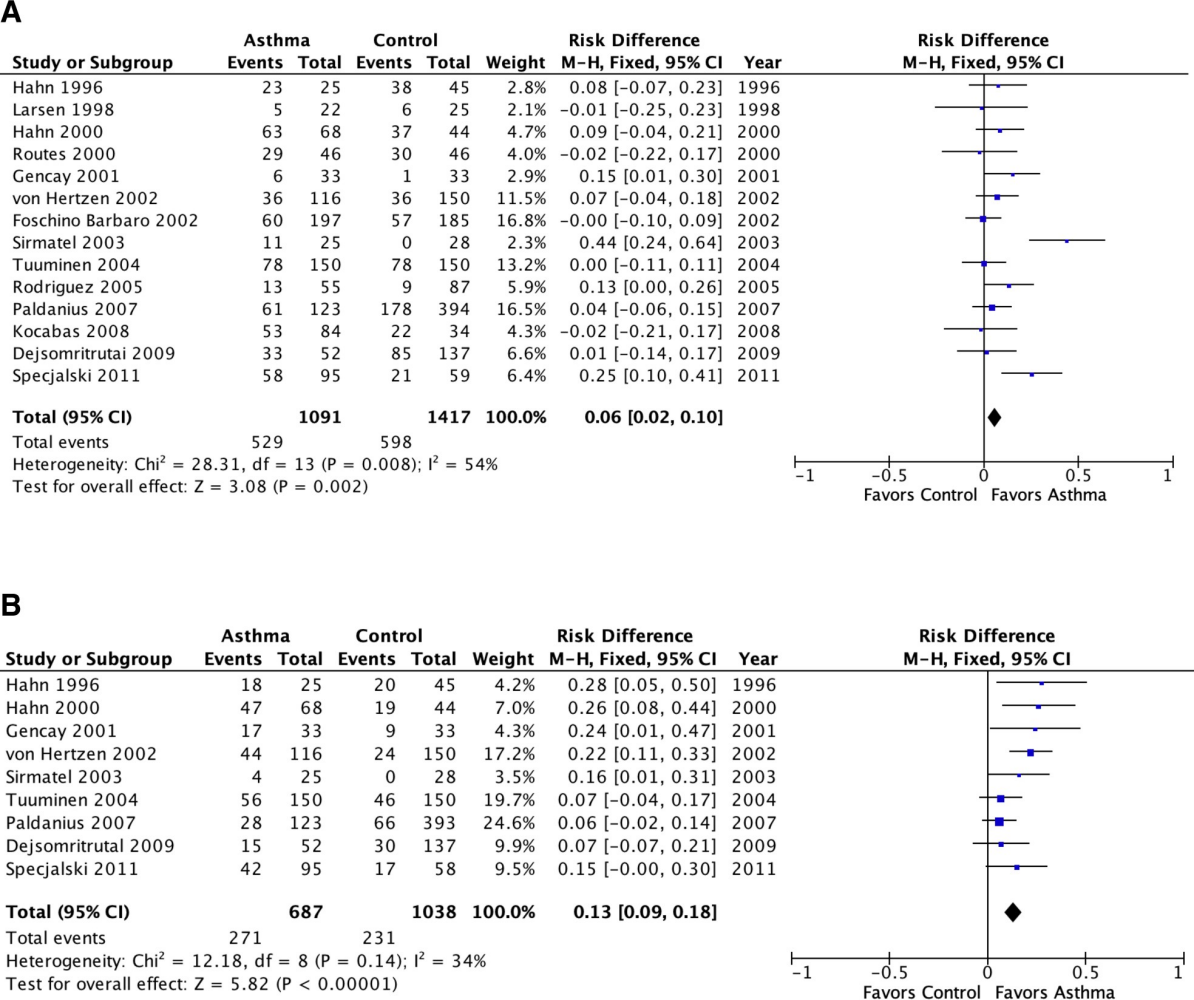
Meta-analysis of population attributable risk (*C*. *pneumoniae* biomarkers and asthma) in adult asthma. A: *C*. *pneumoniae-specific* IgG. B: *C*. *pneumoniae*-specific IgA. Population attributable risk varies from 0 (0%: contributes to none of the disease) to 1 (100%: contributes to all of the disease).

The Cochrane Collaboration provides a formal risk of bias (ROB) assessment tool in RevMan that is appropriate for randomized trials and includes domains such as random sequence generation, allocation sequence concealment, blinding of participants and personnel, blinding of outcome assessment, incomplete outcome data and selective outcome reporting. These domains do not apply to the assessment of case-control studies. For the individual case-control studies included in this meta-analysis four domains were distilled and tailored from the NIH Quality Assessment of Case-Control Studies checklist [10]: (1) *case selection bias* (Are asthma patients with particular characteristics, e.g. smoking and lung co-morbidities, systematically excluded or included?), (2) *case diagnostic certainty* (Is asthma diagnosis supported by objective evidence of reversible airway obstruction or airway hyperreactivity?), (3) *control selection bias* (Are controls representative of the general population?) and (4) *biomarker validity* (How well are the included biomarker tests validated?). Regarding publication bias, the number of studies were generally insufficient to justify using funnel plots to assess for publication bias according to Cochrane Collaboration guidance [11]. A single analysis (Fig 3A) was examined via a funnel plot because it was the only analytic group containing more than 10 studies. Sensitivity analysis was performed for selected analytic groups (Figs 2A, 3A and 5) to investigate subgroup heterogeneity.

## Results

### Updated systematic review

Ten pediatric asthma studies from 7 countries met inclusion criteria (Table 1) [12–21]. Nine studies reported peripheral blood biomarkers (serology in 8, culture in 1) and 1 reported on nasal and/or induced sputum specimens. Five of 10 reported one or more positive associations with asthma (Table 1). Two positive studies included severe asthma patients undergoing bronchoscopy for clinical indications [12,20] and a third positive study reported that biomarker positivity tended to be higher in more severe asthma [17]. Another positive study reported that Cp biomarker positivity was associated with non-atopic asthma [14]. The fifth positive study compared atopic asthma with non-atopic controls, raising the possibility of confounding by atopic status [13]. Of the five studies reporting no significant biomarker association with chronic asthma, one reported a positive association of Cp IgM and acute exacerbations (P = 0.03) [16], and another found a positive association of Cp IgA and asthma in the subgroup with inhalant allergy (P = 0.002) but not in the subgroup with food allergy [18]. A third negative study reported a significant (P<0.001) association of Cp IgG with increased attacks but did not provide primary data [21]. A fourth negative study used as controls a group of patients some of whom had sinusitis, otitis and pneumonia [15] and the fifth negative study did not include severe asthma [19].

**Table 1:** Pediatric case-control studies of Chlamydia pneumoniae biomarkers in asthma.

Reference	Country	Cases	Controls	Microbiologic methods	Main finding(s)	Significant association with asthma? Yes/No	Additional Comments
Webley and coworkers, 2005 [12]	USA	70 patients aged 1 month to 19 years undergoing bronchoscopy for refractory respiratory symptoms (42 w/ asthma, 28 w/ other respiratory conditions).	70 age and sex matched patients without asthma or respiratory conditions.	Blood culture for Cp in all. Bronchoalveolar lavage (BAL) for Cp smear, culture and PCR in cases only	Blood culture for Cp was positive in 20 (34%) of 70 cases v 8 (11%) of 70 controls, p< 0.01; 17 (40.5%) of the asthma subgroup were culture positive. 38 (54%) of 70 cases were Cp PCR+ with no significant differences in Cp prevalence between asthma cases v other respiratory illness cases.	Yes	Cp was detected in both asthma and in subjects with poorly differentiated chronic respiratory illnesses. Of the 42 asthma patients, 28 (67%) were BAL PCR positive and 14 of these were also positive on culture, indicating a high prevalence of active and/or persistent infection. Elevated total IgE was present in 20 (48%) of 42 subjects with positive BAL cultures compared to 9 (19%) of 48 with negative BAL cultures, p< .0001, suggesting a relationship between infection and atopy.
Kopriva and coworkers, 2005 [13]	Czech Republic	149 atopic children ages 4–8 with chronic cough (83) or asthma (66).	241 non-atopic children with non-respiratory complaints.	Serology: IgM, IgG, IgA by ELISA (Medac, Hamburg, Germany). Cutoff values for seropositivity not provided.	Seropositivity (IgG and IgM or IgG and IgA) positivity was 49 (33%) of 149 cases v 38 (16%) of 241 controls, p< 0.01. Seropositivity was 29 (44%) of 66 in the asthma subgroup, p< 0.0001.	Yes	The case subgroup with asthma had mild asthma. The fact that cases were atopic and controls were non-atopic raises the issue of confounding by atopic status.
Teig and coworkers, 2005 [14]	Germany	38 children ages 7–15 with chronic stable lung disease (26 with asthma, 12 with chronic purulent bronchitis).	42 healthy children ages 6–15.	Nested PCR on nasal brush specimens and/or induced sputum	Cp PCR was positive in 9 (24%) of 38 cases v 0 of 42 controls, p = 0.001). When analyzed separately, both asthma and chronic bronchitis were significantly associated with PCR positivity (4/26 (15.4%) and 5/12 (41.6%) v 0/42, p = .017 and < 0.001, respectively).	Yes	Case status was also associated with *M*. *pneumoniae* (Mp) PCR positivity (4 (11%) of 38 cases v 0 controls, p< .05). Significantly more children with non-atopic asthma than with atopic asthma were Cp PCR positive and/or Mp PCR positive (4/8 v 1/18, p = .02). No correlation was found between Cp detection and severity of lung disease.
Dal Molin and coworkers, 2005 [15]	Italy	47 children ages 5–12 with ever-asthma, part of a population-based survey	180 children ages 5–12 without ever-asthma, part of a population-based survey	Serology: IgG and IgA by MIF on sera that screened positive by a commercial ELISA (Elegance, Bioclone Australia)	Cp IgG seroreactivity was detected in 8 (17.0%) of 47 cases v 180 (29.9%) of 602 controls, p = 0.09. Cp IgA seroreactivity was detected in 5 (10.6%) of 47 cases v 123 (20.4%) of 602 controls, p = 0.15.	No	The control group contained subjects with respiratory illnesses, e.g., sinusitis (18), otitis (54), pneumonia (7).
Annagur and coworkers, 2007 [16]	Turkey	79 asthmatic children and adolescents ages 5–15 years (37 with acute exacerbations, 42 with stable asthma).	36 non-atopic healthy children and adolescents.	Serology: specific IgM and IgG by ELISA (IBL, Hamburg. Germany)	Cp IgG positivity was found in 17 of 42 (40.5%) stable asthma cases v 8 (22%) of 36 controls, p = 0.07.	No	Cp IgM indicative of acute infection was significantly associated with acute asthma exacerbations (5 (14%) of 37 v 0 with stable asthma v 0 controls, p< 0.01). *M*. *pneumoniae* IgM was also associated with exacerbations (8% v 0% controls, p = .031). There were no associations with H. pylori antibodies.
Wazir and coworkers, 2007 [17]	India	54 children ages 5–14 with physician-diagnosed chronic stable persistent asthma (26 mild-, 20 moderate-, 8 severe-persistent)	34 healthy children of similar age.	Serology: specific IgG by ELISA (Savyon, Israel)	Cp IgG positivity was found in 22 (41%) of 54 cases of chronic stable asthma v 4 (11%) of 34 controls, p = 0.004)	Yes	IgG positivity was somewhat more common in moderate and severe persistent- compared to mild-persistent asthma (14 (50%) of 28 mod-severe persistent asthma v 8 (31%) of 26 mild persistent asthma, p = .18).
Nagy and coworkers, 2007 [18]	Hungary	141 patients with asthma aged 3–21 years	(1) 125 healthy controls aged 3–12 (2) 62 allergic but non-asthmatic patients aged 4–20 years.	Serology: IgG & IgA by ELISA	Cp IgG positivity was found in 61 (43.2%) of 141 cases of chronic stable asthma v 90 (48.1%) of 187 controls, p = 0.45. Cp IgA positivity was found in 45 (31.9%) of 141 cases of chronic stable asthma v 61 (32.6%) of 187 controls, p = 0.91.	No	There were no patients with severe persist asthma in the study group. Irrespective of case/control status, no food/drug-allergic patient was positive for Cp IgA, whereas 41.6% of the inhalant allergic patients were positive (p = 0.002).
Kazar and coworkers, 2011 [19]	Czech Republic	97 children and adolescents with stable mild to moderate persistent asthma.	129 children and adolescents with no respiratory illness or asthma.	Serology: specific IgA and IgG by ELISA (Savyon, Israel)	Cp IgG positivity was found in 19 (19.6%) of 97 cases v 26 (20.1%) of 129 controls, p = 0.88). Cp IgA positivity was found in 14 (14.4%) of 97 cases v 18 (13.9%) of 129 controls, p = 0.94).	No	This study did not include severe persistent asthma.
Patel and coworkers, 2012 [20]	USA	177 pediatric patients with severe inflammatory lung diseases, unresponsive to corticosteroids, of whom 143 had asthma.	35 healthy age- and gender-matched controls.	Serology: Cp-specific IgE by immunoblotting	Cp-specific IgE was detected in 97 (55%) of 177 cases v 0 (0%) of 35 healthy controls (P = 0.0001).	Yes	The study did not report asthma separately. All cases underwent bronchoscopy: 100 (56%) of 177 cases were Cp PCR positive in BAL. Cp IgE was also detected in BAL.
Tutanç and coworkers [21]	Turkey	66 pediatric patients with bronchial asthma, ages 3 to 14	46 healthy children of similar ages	Serology: Cp IgG by indirect immunofluorescence (EUROIMMUNE)	Cp-specific IgG was detected in 18 (27.3%) of 66 cases v 6 (13.0%) of 46 healthy controls (P = 0.10).	No	Cp IgG seropositivity was reported as significantly (P<0.001) higher in patients with more frequent attacks.

Ten adult asthma studies from 8 countries met inclusion criteria (Table 2) [22–31]. Eight studies reported peripheral blood biomarkers (all serology) and 2 reported on nasal and/or induced sputum specimens. Five of 10 studies reported one or more positive associations with asthma (Table 2). Two positive studies reported significant associations of Cp biomarkers and asthma severity [30] or poor control [29] while the three other positive studies did not document the level of asthma severity/control. One negative study found a significant association of Cp PCR positivity and lower FEV1/FVC (P = 0.02) [24]. Another negative study used family members as controls instead of general community members [26]. In two other negative studies asthma was mild [25] or did not require active symptoms [28]. The fifth negative study failed to include a full array of biomarker data [22].

**Table 2:** Adult case-control studies of *Chlamydia pneumoniae* biomarkers in asthma.

Reference	Country	Cases	Controls	Microbiologic methods	Main finding(s)	Significant association with asthma? Yes/No	Additional Comments
Park and coworkers, 2005 [22]	Korea	36 adults with stable asthma	45 controls without asthma or COPD (unknown clinical status).	Serology: IgM & IgG by MIF	Cp IgG >1:512 or IgM >1:20 was 3 (8.3%) of 36 cases v 1 (2.2%) of 45 controls, P = 0.2)	No	No biomarker data for lesser degrees of Cp seroreactivity were provided.
Rodriguez and coworkers, 2005 [23]	Spain	55 adults with intrinsic asthma	87 healthy blood donors.	Serology: IgG by LPS rELISA (Medac, Hamburg, Germany)	Cp IgG OD>1.1 (seropositivity threshold) was detected in 13 (24%) of 55 cases v 9 (10%) of 87 controls, p = 0.033).	Yes	Article in Spanish.
Harju and coworkers, 2006 [24]	Finland	103 adult asthma patients (53 mild, 50 moderate)	30 controls without respiratory complaints and with normal lung function.	PCR from NP swabs and induced sputum	Cp PCR+ was detected in 10 (19%) of 53 mild and 8 (16%) of 50 moderate asthma cases v 9 (30%) of 30 controls, p = 0.304.	No	PCR positive asthmatics had lower FEV1/FVC than PCR negative cases (78% v 81%, p = .023).
Paldanius and coworkers, 2007 [25]	Finland	123 military recruits with asthma	394 military recruits without asthma	Serology: IgG & IgA by MIF	At entry to military duty: Cp IgG ≥1:32 was detected in 61 (49.6%) of 123 cases v 178 (45.3%) of 394 controls, p = 0.45. Cp IgA ≥1:10 was detected in 28 (22.8%) of 123 cases v 66 (16.8%) of 393 controls, p = 0.17.	No	Severity of asthma was not documented. It was presumably of mild degree to allow for military duty.
Kocabas and coworkers, 2008 [26]	Turkey	84 adults with stable asthma (32 mild intermittent, 38 mild persistent, 13 moderate persistent, 1 severe persistent).	34 healthy adult relatives of the cases.	Serology: IgM & IgG by MIF Cp PCR of throat wash	IgG≥1:16 was detected in 53 (63.1%) of 84 cases v 22 (64.7%) of 34 controls, P = NS. Cp PCR was detected in 24 (29%) of 84 cases v 4 (12%) of 34 controls, p = 0.059.	No	Choice of family members instead of general community members as controls may have contributed to similar seroprevalence across groups. No serologic acute infections were noted for any PCR+ subject, suggesting chronic infection.
Torshizi and coworkers, 2008 [27]	Iran	20 adults with chronic stable asthma.	20 pair-matched healthy controls.	Cell culture (McCoy cells) of nasal epithelial cells obtained from nasopharyngeal swabs	Positive culture was detected in 7 (35%) of 20 stable asthma cases v 1 (5%) of 20 matched controls, P = 0.044.	Yes	Positive culture was also detected in 10 (48%) of 21 patients with asthma exacerbations v 3 (14%) of 21 pair-matched controls, P = 0.043
Dejsomritrutai and coworkers, 2009 [28]	Thailand	52 adults (20–44 years old) with asthma (reversible airway obstruction and asthma symptoms within the past year) identified from a population-based cohort study.	137 adults without bronchial hyperreactivity BHR or asthma, matched by age and geographic location and randomly selected from the same cohort study.	Serology: IgM, IgG & IgA by MIF	IgM ≥1:10: 12 (23.1%) of 52 cases v 20 (14.6%) of 137 controls, p = 0.17. IgG≥1:32: 33 (63.5%) of 52 cases v 85 (62.0%) of 137 controls, p = 0.86. IgA ≥1:32: 15 (28.8%) of 52 cases v 30 (21.9%) of 137 controls, p = 0.32.	No	Severity of asthma was not documented, and did not require current symptoms.
Specjalski and coworkers, 2011 [29]	Poland	95 patients with persistent asthma	58 healthy controls	Serology: Cp-specific IgG & IgA by ELISA (Savyon) PCR in induced sputum (subset)	Cp IgG seroreactivity was detected in 58 (61.1%) of 95 cases v 21 (36.2%) of 58 controls, p<0.005. Cp IgA seroreactivity was detected in 42 (44.2%) of 95 cases v 17 (29.3%) of 58 controls, p = 0.09.	Yes	IgG was detected more frequently in patients with uncontrolled (p = 0.001) and nonatopic (p<0.05) asthma. Cp PCR was positive in 8 (40%) of 20 asthma patients.
Hahn and coworkers, 2012 [30]	USA	66 outpatients with asthma.	51 platelet donors	Serology: Cp-specific IgE by immunoblotting against Cp EB proteins Cp PCR of whole blood (cases only)	Cp IgE seroreactivity was detected in 33 (50%) of 66 cases v 4 (7.8%) of controls, p<0.0001.	Yes	Cp IgE seroreactivity was detected in 4 (21%) of 19 mild intermittent, 4 (67%) of 6 mild persistent, 14 52%) of 27 moderate persistent and 11 (79%) of 14 severe persistent asthma cases, p = 0.002 for trend. Cp PCR was detected in 25% of asthma subjects, and was strongly associated with the presence of Cp IgE (P = 0.001).
Smith-Norowitz and coworkers, 2020 [31]	USA	22 Cp IgG seropositive asthma patients enrolled from an inner-city primary care clinic	22 Cp IgG seropositive non-asthma controls enrolled from an inner-city primary care clinic	Serology: Cp IgE by a modification of an enzyme immunoassay (EIA) (Labsystems, Vantaa, Finland) using Cp OMP as the antigen	Cp IgE was detected in 21 (95.5%) of 22 cases v 10 (45.5%) of 22 controls, p = 0.0006.	Yes	Severity of asthma was not documented.

No prospective studies meeting inclusion criteria were found. One prospective study in adults was derived from an historical cohort organized for other purposes and failed to meet case definition criteria for asthma [32]. Three pediatric studies followed small numbers of children for less than 2 years and failed to meet duration criterion [33–35]. Thus, there were no long-term prospective studies designed specifically to investigate Cp infection in asthma.

### Meta-analysis

The meta-analyses included 11 studies published prior to 2005 [36–46] that were tabulated in the 2005 review [8] and not re-tabulated, and 14 studies published since 2005 [15–21,23,25,26,28–31] that are presented in Tables 1 and 2. Fig 2 presents meta-analyses of the PARs of Cp IgG and Cp IgA for pediatric asthma. There were no significant associations with asthma for either antibody in pediatric asthma (i.e., the PAR confidence intervals include 0%). For IgG there appeared to be significant underlying heterogeneity (I^2^ = 76%, P = 0.0001). The heterogeneity for Cp IgG is completely explained by three outliers: I^2^ became zero when Wazir 2007 [17], Annagur 2007 [16] and Tutanç 2015 [21] were removed from the meta-analysis. Wazir 2007 [17] contained almost two-thirds of patients with moderate to severe asthma and was the only one of the studies in Fig 2A to specify asthma severity. The contribution of asthma severity category to the heterogeneity is explored further in the analysis depicted in Fig 5.

Fig 3 presents comparable results for adult asthma. Cp IgG was associated with a significant but small PAR (6%, 95% confidence interval 2%-10%, p = 0.002). Cp IgA was associated with a significant somewhat larger PAR (13%, 9%-18%, p<0.00001). Both estimates exhibited a moderate amount of statistical heterogeneity (I^2^ = 54% and 34%, respectively). Similar to the case for children, the heterogeneity for Cp IgG is completely explained by outliers: I^2^ became zero when Sirmatel 2003 [44] and Specjalski 2001 [29] were removed from the meta-analysis. In Specjalski 2001 [29] almost three-quarters of the patients had moderate to severe asthma. Sirmatel 2003 [44] did not provide asthma severity data. A funnel plot identifies these two studies as lying outside the funnel (S1 Fig). This asymmetry is not necessarily related to publication bias; rather it may possibly be explained by a greater than average severity, as explored further in analyses associated with Fig 5.

**Fig 4:**
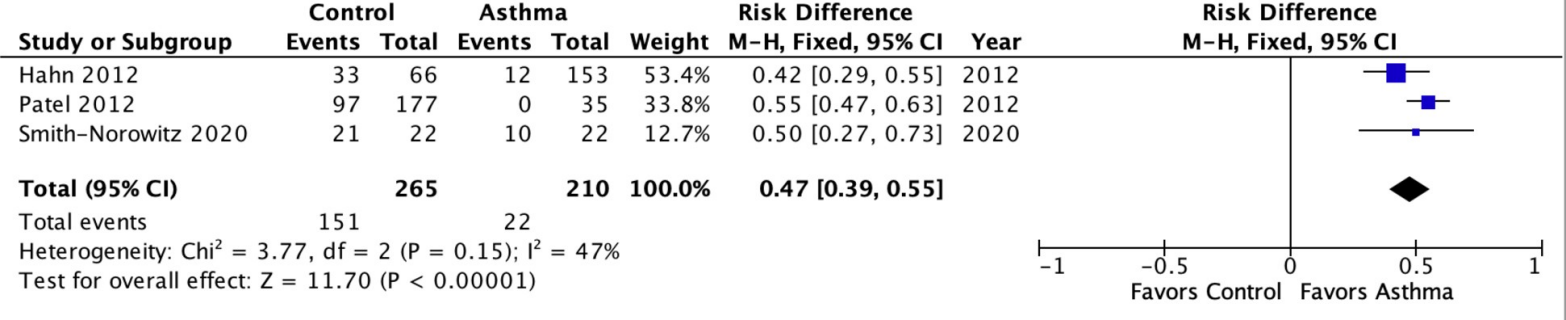
Meta-analysis of population attributable risk for *C*. *pneumoniae-specific* IgE in pediatric and adult asthma combined. Pediatric asthma: Patel 2012. Adult asthma: Hahn 2020 & Smith-Norowitz 2020. Population attributable risk varies from 0 (0%: contributes to none of the disease) to 1 (100%: contributes to all of the disease).

Fig 4 presents results for Cp IgE for combined age groups. In contrast to the IgG and IgA results, the PAR for IgE was large (47%, 39%-55%, p<0.00001) with only moderate heterogeneity (I^2^ = 47%, P = 0.05) despite combining child and adult asthma.

**Fig 5:**
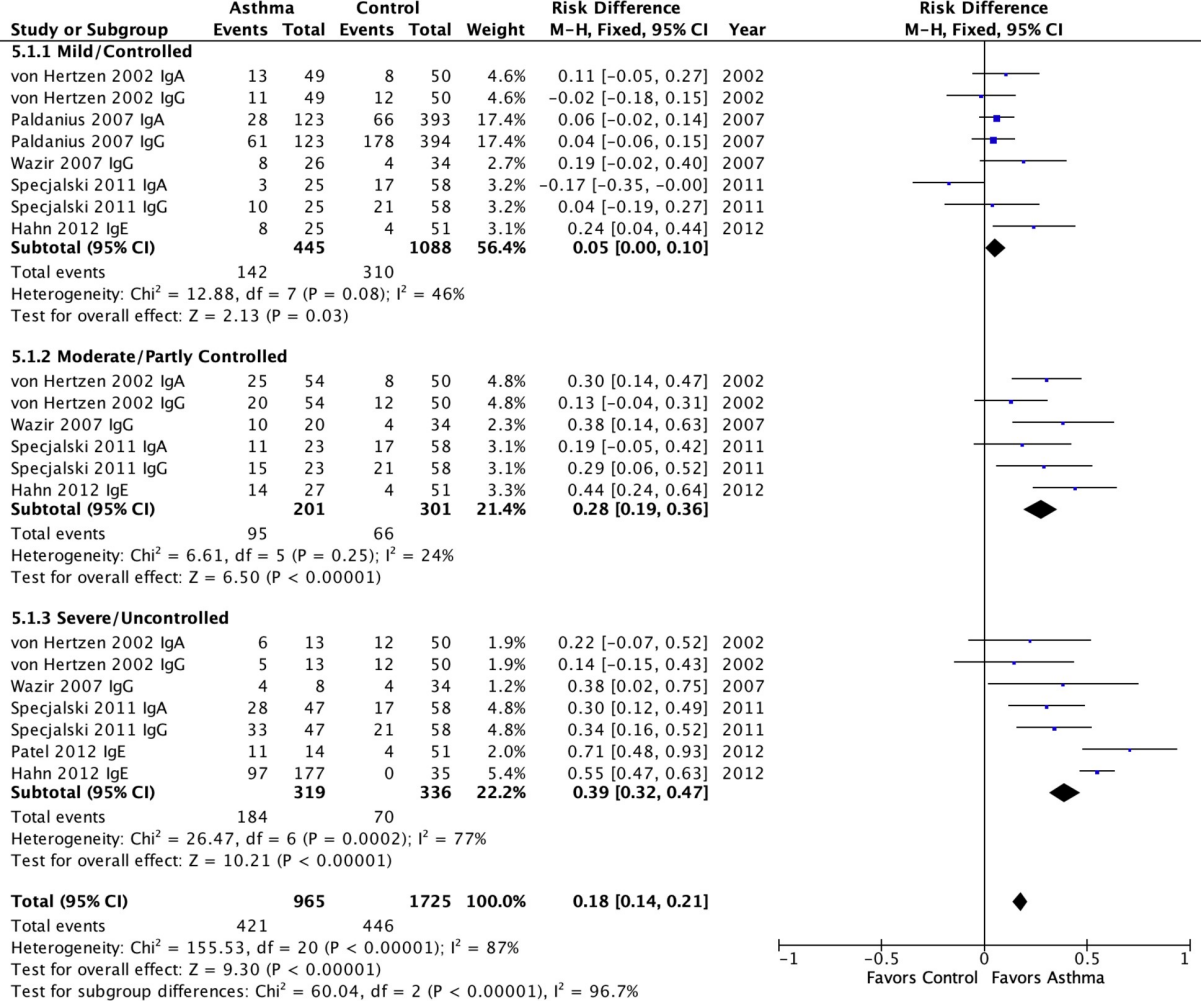
Meta-analysis of population attributable risk for *C*. *pneumoniae-specific* IgG, IgA & IgE in pediatric and adult asthma by severity subgroups. Pediatric asthma: Patel 2012. Adult asthma: All others. Population attributable risk varies from 0 (0%: contributes to none of the disease) to 1 (100%: contributes to all of the disease).

Fig 5 presents the meta-analytic results for studies that reported on asthma severity categories. This analysis combined asthma age groups and used all available antibody results (IgG, IgA, IgE). There was a strong positive association of Cp biomarker positivity and asthma severity category that was highly significant (P<0.00001). Cp biomarkers were associated with a 5% PAR (0%-10%, p = 0.03) in mild/controlled asthma, a 28% PAR (19%-36%, p<0.00001) in moderate/partly controlled asthma and a 39% PAR (32%-47%, p<0.00001) in severe/uncontrolled asthma. As one might predict when combining age groups and isotypes, within subgroup heterogeneity ranged from moderate (I^2^ = 46%) for the mild/controlled subgroup to substantial (I^2^ = 77%) for the severe/uncontrolled subgroup. The exception was the moderate/partly controlled subgroup that exhibited negligible (I^2^ = 24%) heterogeneity. Importantly, the PARs between subgroups were highly significantly different (p<0.00001), confirming that severity was likely responsible for some of the heterogeneity noted in previous results.

Interestingly, when Cp IgE studies are removed from the meta-analysis (S2 Fig), the heterogeneity for Moderate/Partly Controlled and Severe/Uncontrolled categories disappears (I^2^ = 0). Nevertheless, the significant associations of severity category and the other Cp biomarkers remain significant (P<0.00001), despite the fact that the PARs diminish from 28% to 25% for Moderate/Partly Controlled and from 39% to 29% for Severe/Uncontrolled asthma. These patterns suggest that the associations are robust across biomarker types and that Cp IgE produces the strongest associations.

### Study quality

Quality assessment results for the studies included in the meta-analyses are presented in supplementary file S2 Appendix. Case selection was rated as good in a minority (5 of 12) of adult asthma studies that could be assessed, indicating that external validity was adversely affected by exclusion of patients who smoked and/or had lung comorbidities. An additional 4 adult studies failed to provide enough information to allow a rating, an indication of inadequate reporting quality. The majority of studies (15 of 23) confirmed the asthma diagnosis with objective measures and only 2 studies failed to provide data, indicating that diagnostic certainty was generally good. Most (n = 17) control groups were composed of convenience samples of a healthy (usually patient) population. Only 3 studies reported choosing controls randomly and 5 studies were rated as having made poor choices of controls on the basis of using family members, health care workers, or using inappropriate matching criteria (e.g., intentional mismatches between atopic and non-topic cases and controls). Biomarker validity was rated as Good for 15 studies that employed a version of the microimmunofluorescence (MIF) test or other tests that had been validated against organism detection in either that study or other publications. Nine studies employed an ELISA test of unknown validity and one study did not provide any test information.

## Discussion

This systematic review/meta-analysis focused on chronic asthma, not asthma exacerbations, and was undertaken to update observational studies published since 2005, the date of the last review [8] and, for the first time, to estimate the population attributable risk (PAR) of Cp biomarkers in chronic asthma. Studies of short-term azalide/ketolide treatment for asthma exacerbations have been disappointing [47,48] whereas studies of long-term macrolide/azalide treatment for long-term management of chronic asthma have yielded clinically significant benefits [49], hence the focus on chronic asthma herein. Underlying mechanism(s) of action for macrolide benefits in chronic asthma are unknown and may include pathogen and host-directed anti-inflammatory effects [50]. The meta-analyses found nul to small associations of Cp IgG and IgA with asthma in children and adults, respectively. These results are not surprising given that children often do not mount a detectable antibody response in proven Cp infection [12] and the background prevalence of Cp antibody in the adult population is high [51]. These results suggest that Cp IgG and IgA may not prove clinically useful in predicting which patients will benefit from macrolide treatment. Further analysis found that Cp IgE population attributable risk approached 50% (Fig 4). This result suggests that Cp chronic infection cannot be discounted as a potentially significant contributing factor to asthma, particularly moderate to severe asthma, in both children and adults.

Population attributable risk (PAR) as an outcome was chosen over the odds ratio (OR) because PAR is more clinically interpretable. Patterns and significance of results did not change when OR was substituted as an outcome measure in each meta-analysis (data not shown). Neither Cp IgG nor IgA had a significant association with PAR in child asthma, and had only modest associations (6% and 13%, respectively) in adult asthma. Cp IgG indicates past infection, which is prevalent in adult populations worldwide [51]. Cp IgA is thought to better reflect possible ongoing chronic infection [39]. This study did not assess the value of combined biomarkers (e.g. IgG≥1:xxx and IgA≥1:xx). By contrast with IgG and IgA results, the PAR for Cp IgE was a surprisingly high 47% (95% CI 40% to 55%) with little heterogeneity despite combining asthma age groups. The pathogenicity of antigen-specific IgE in asthma is well established and antigen avoidance and/or removal is the foundation of asthma symptom prevention and treatment. Asthma, whether atopic or non-atopic (i.e., associated or not with skin test positivity against a battery of aeroallergens) is almost always associated with some type of IgE-related reaction [52]. The results of this meta-analysis raise the possibility that some undetected antigen(s) responsible for generating a cryptic IgE response in some asthma patients could be expressed by *Chlamydia pneumoniae*.

Whether Cp IgE reflects ongoing Cp infection or a “hit and run” phenomenon is not established. Bronchoscopic studies of severely symptomatic children with asthma and/or chronic bronchitis have demonstrated the presence of Cp (by PCR, staining and/or culture) in around half the studied patients [12,20,53]. One of these studies reported that elevation of total IgE was strongly associated with lavage culture positivity of *Chlamydia* [12]. These observations suggest that Cp IgE should be explored further for its accuracy in predicting the presence of ongoing treatable chronic Cp infection in asthma. There are no comparable bronchoscopic studies of adults with severe asthma. Cp has been detected within lung tissue monocytes by immunostaining in 44% of young adults (mean age 32 years) autopsied after traumatic deaths [54] and Cp PCR positivity in peripheral blood is equally prevalent in healthy blood donors [55,56]. Thus, chronic exposure to Cp antigens appears to be common. Perhaps the presence of Cp IgE in asthma reflects an aberrant host immune response that is causally related to reactive airways diseases. Cp IgM, IgG and IgA are the commercially available biomarkers used to assess acute Cp infection in medical practice. The results of this study suggest that these commercially available tests have limited sensitivity and specificity to identify potentially chronically infected individuals. Cp IgE may be a more promising biomarker to explore in patients with asthma.

Severe asthma is currently a guideline criterion for macrolide treatment [1–3]. Fig 5 presents evidence that Cp biomarkers, whether IgG, IgA or IgE, are associated with increasing asthma severity in a somewhat linear fashion. This finding has two implications: (1) Cp infection is a potential underlying cause for macrolide benefits in a subset of patients with severe asthma and (2) some proportion of less than severe asthma may also be attributable to infection. It has not been ruled out that macrolides (and perhaps other antibiotics, or combinations of antibiotics) are beneficial for some selected patients with moderate/partly controlled or even mild/controlled, asthma. Based on the data in Fig 5, the estimated numbers needed to treat (NNT) are 20 for mild/controlled asthma, 4 for moderate/partly controlled asthma, and 3 for severe/uncontrolled asthma. These estimates assume that antibiotics are completely effective in removing the risk factor and that the risk factor is causal or a necessary contributing factor. The former assumption is unlikely to be true and the latter is plausible but unproven. The results do support the need however to include Cp biomarkers (especially Cp IgE) in future macrolide treatment trials, and to include less than severe asthma in such trials. Clinicians should keep in mind the possibility that they are treating an infection as they prescribe macrolides for asthma, and consider following a recommendation of the British Thoracic Society guidelines [3] that “ongoing treatment should be guided by clinical response based on specific outcome measures, including exacerbation frequency, symptoms and quality of life assessed at baseline.”

### Limitations

This review and meta-analysis did not address a potential role for *Mycoplasma pneumoniae* (Mp). Mp is associated with asthma exacerbations, mostly but not exclusively in children [57], and has recently been associated with asthma inception in a population-based study [58]. This review and meta-analysis did not address the considerable amount of *in vitro* and *in vivo* experimental data supporting the plausible role for Cp in asthma pathogenesis, extensively reviewed elsewhere [59–61]. This study has limitations including issues with diversity of diagnosis, patient selection, and study and biomarker methodology in the source material, all of which may contribute to analytic heterogeneity (i.e., the possibility that an analysis is missing important subgroup differences and/or comparing “apples and oranges”). The main requirement for inclusion in this study was clinician-diagnosed asthma. Some, but not all, studies included objective evidence for reversible airway obstruction. Up to 30% of physician diagnosed asthma patients may not have the diagnosis confirmed after systematic evaluation [62]. Overdiagnosis would tend to decrease contrasts, i.e., bias towards nul findings and is less likely as asthma severity increases [62]. Variations in choice of the control group likely increased heterogeneity and probably also biased towards the nul. For example, Kocabas et al. [26] used relatives of cases as controls, which probably biased results towards the nul because family members are likely to share viruses and bacteria. Another source of bias is the all too common practice in asthma case-control studies to conflate asthma and atopy by comparing, for example, an atopic case group with a non-atopic control group (or vice versa), as was done by Kopriva et al. [13]. Variability in biomarker methodology included differences in laboratory test methods and use of different criteria for positive and negative results. This variability underscores the fact that there are no established serological criteria for chronic infection [63]. All these limitations likely contributed to the heterogeneity of results. Despite many of these limitations biasing towards the nul the results for Cp IgE and severity were quantitatively large. An assessment of the prevalence of these biases can be found in the supplementary S2 Appendix.

### Study quality assessment

The Cochrane Collaboration has developed a tool for Risk of Bias (ROB) assessment of randomized trials of medical interventions but has not developed a quality tool for case-control studies. Therefore, the case-control studies included in the meta-analyses reported here were assessed using a tool developed for this report that included four domains relevant to the assessment of case-control studies: case selection bias, case diagnostic certainty, control selection bias and biomarker validity. *Case selection bias* assessed the extent to which the included cases were representative of typical asthma patients in the population, as a measure of generalizability or external validity. Asthma efficacy trials upon which the guideline treatments are based systematically exclude asthma patients who smoke, or who have ever smoked more than 10 pack-years, or who suffer from lung co-morbidities such as chronic bronchitis and/or emphysema [64,65]. These types of selection bias are critically important to acknowledge because biomarkers of *C*. *pneumoniae* infection are associated with smoking, chronic bronchitis and emphysema [7,66,67]. The case selection bias domain qualitatively assessed the degree to which such exclusions were implemented in individual case-control studies: a Good rating was given to studies that included smoking and co-morbidities, a Fair rating was given to studies that included one but not both and a Poor rating was given to studies that excluded both smoking and lung co-morbidities. The *case diagnostic certainty* domain assessed whether the asthma diagnosis was supported by objective evidence of either reversible airway obstruction measured by pulmonary function testing or bronchial hyperreactivity testing. Clinician diagnosed asthma–the primary inclusion criterion in this report–may be incorrect in up to 30% of diagnosed patients [62]. Such overdiagnosis is more common in less severe asthma and may decrease power to show case-control differences. A Good rating was applied to studies that included systematic objective airway testing and a Fair rating was given to studies without such systematic testing. *Control selection bias* assessed the extent to which the included controls were representative of typical individuals without asthma in the general population. Choosing randomly selected healthy individuals received a Good rating. Selecting a convenience sample of healthy non-asthma controls was given a Fair rating. Selecting family members, health care workers or subjects with respiratory illnesses as controls received a Poor rating because transmission and/or prevalence of *C*. *pneumoniae* infection is enhanced in these settings [59]. *Biomarker validation* assessed the extent to which the serologic test(s) employed had been validated against human infections documented by organism detection. A Good rating was given, e.g., to the microimmunofluorescence (MIF) test that has been assessed against human population infections confirmed by organism detection [68]. A Fair rating was given to enzyme-linked immunosorbent assays (ELISAs) or other serological tests for which no comparable published validation data could be found. Overall quality assessment results confirm the need for larger, better designed studies into the potential role of chronic *C*. *pneumoniae* infection in asthma. Ideally such studies should include direct organism identification from the lung but this ideal appears infeasible currently and thus indirect biomarkers of infection such as serology will be required pending advances in chlamydial sampling and diagnostics for chronic lung infection.

Publication bias is a recognized significant issue for reports of randomized trials of interventions. It is not clear that publication bias affects the reporting of case-control studies to a comparable extent because there are few if any financial disincentives to reporting negative results of case-control studies compared to randomized trials of medical treatments. Except for a single funnel plot associated with Fig 3A, no formal assessment of publication bias was possible for this report due to the limited number of published studies. It is worth noting the paucity of any type of study into this potentially important area of inquiry [69]. In particular, this review highlights the lack of any long-term, population-based prospective observational studies of *C*. *pneumoniae* infection in asthma inception or severity.

In summary, this updated systematic review found that biomarkers of *Chlamydia pneumoniae* infection are associated with chronic asthma in half of observational studies and heterogeneity was suggested based on asthma age group, disease severity category and/or antibody isotype. Meta-analysis results confirmed that age (child asthma v adult asthma), severity (mild/controlled, moderate/partly controlled, severe/uncontrolled) and isotype (IgG, IgA, IgE) were all significantly associated with heterogeneous population attributable risks for asthma. Results also suggest that potential “bacterial allergy” (i.e., presence of Cp-specific IgE) may be a quantitatively important pathogenetic mechanism in asthma, particularly in moderate/partly controlled and severe/uncontrolled asthma. These findings should inform future research into macrolide mechanisms of action in asthma and may inform clinicians’ macrolide treatment plans for selected patients.

## Supporting information

S1 Checklist PRISMA 2009 checklist. (DOC)

S1 Fig Funnel plot of 14 studies analyzed in Fig 3A. (DOCX)

S2 Fig Results of sensitivity analysis of severity subgroups (Fig 5) with Cp IgE seroreactivity results removed. (DOCX)

S1 Appendix Search algorithms for updated systematic review. (DOCX)

S2 Appendix Quality assessment table for case-control studies included in the meta-analyses. (DOCX)

S1 File Research in context. (DOCX)

S1 Dataset Minimal data set. (PDF)

## References

[1] GINA. Difficult-to-Treat and Severe Asthma in Adolescent and Adult Patients: Diagnosis and Management. 2019;https://ginasthma.org/wp-content/uploads/2019/04/GINA-Severe-asthma-Pocket-Guidev2.0-wms-1.pdf, accessed June 20, 2020.

[2] Holguin F, Cardet JC, Chung KF, Diver S, Ferreira DS, Fitzpatrick A, et al. Management of severe asthma: a European Respiratory Society/American Thoracic Society guideline. Eur Respir J. 2020;55(1). Epub 2019/09/29. 10.1183/13993003.00588-2019 . 31558662

[3] Smith D, Du Rand IA, Addy C, Collyns T, Hart S, Mitchelmore P, et al. British Thoracic Society guideline for the use of long-term macrolides in adults with respiratory disease. BMJ Open Respir Res. 2020;7(1). Epub 2020/04/26. 10.1136/bmjresp-2019-000489 32332022 PMC7204798

[4] Wong EH, Porter JD, Edwards MR, Johnston SL. The role of macrolides in asthma: current evidence and future directions. Lancet Respir Med. 2014;2(8):657–70. 10.1016/S2213-2600(14)70107-9 . 24948430

[5] Grayston JT, Kuo C-C, Wang S-P, Altman J. A new Chlamydia psittaci strain, TWAR, isolated in acute respiratory tract infections. NEJM. 1986;315(3):161–8. 10.1056/NEJM198607173150305 3724806

[6] Hahn DL, Dodge R, Golubjatnikov R. Association of Chlamydia pneumoniae (strain TWAR) infection with wheezing, asthmatic bronchitis and adult-onset asthma. JAMA. 1991;266:225–30. PubMed Central PMCID: PMC2056624. 2056624

[7] Hahn DL. *Chlamydia pneumoniae*, asthma and COPD: what is the evidence? Ann Allergy Asthma Immunol. 1999;83:271–92. 10.1016/S1081-1206(10)62666-X 10541419

[8] Johnston SL, Martin RJ. *Chlamydophila pneumoniae* and *Mycoplasma pneumoniae*. A role in asthma pathogenesis? Am J Respir Crit Care Med. 2005;172:1078–89. 10.1164/rccm.200412-1743PP 15961690

[9] Uter W, Pfahlberg A. The application of methods to quantify attributable risk in medical practice. Stat Methods Med Res. 2001;10(3):231–7. Epub 2001/07/12. 10.1177/096228020101000305 . 11446150

[10] NIH. Quality Assessment of Case-Control Studies. https://wwwnhlbinihgov/health-topics/study-quality-assessment-tools, accessed January 10, 2020.

[11] Page MJ, Higgins JPT, Sterne JAC. Chapter 13: Assessing risk of bias due to missing results in a synthesis. In: Higgins JPT, Thomas J, Chandler J, Cumpston M, Li T, Page MJ, Welch VA (editors). Cochrane Handbook for Systematic Reviews of Interventions version 6.1 (updated September 2020). Cochrane, 2020. Available from www.training.cochrane.org/handbook.

[12] Webley WC, Salva PS, Andrzejewski C, Cirino F, West CA, Tilahun Y, et al. The bronchial lavage of pediatric patients with asthma contains infectious Chlamydia. Am J Respir Crit Care Med. 2005;171(10):1083–8. 10.1164/rccm.200407-917OC . 15735056

[13] Kopriva F, Szotkowska J, Zapalka M. Bronchial asthma and Chlamydia pneumoniae antibodies in children aged 4–8 years in Olomouc district. Biomed Pap Med Fac Univ Palacky Olomouc Czech Repub. 2005;149(2):289–91. Epub 2006/04/08. 10.5507/bp.2005.044 . 16601774

[14] Teig N, Anders A, Schmidt C, Rieger C, Gatermann S. Chlamydophila pneumoniae and Mycoplasma pneumoniae in respiratory specimens of children with chronic lung diseases. Thorax. 2005;60:962–6. 10.1136/thx.2005.041004 16143584 PMC1747249

[15] Dal Molin G, Longo B, Not T, Poli A, Campello C. A population based seroepidemiological survey of Chlamydia pneumoniae infections in schoolchildren. J Clin Pathol. 2005;58(6):617–20. Epub 2005/05/27. 10.1136/jcp.2004.024380 15917413 PMC1770689

[16] Annagur A, Kendirli SG, Yilmaz M, Altintas DU, Inal A. Is there any relationship between asthma and asthma attack in children and atypical bacterial infections; *Chlamydia pneumoniae*, *Mycoplasma pneumoniae* and *Helicobacter pylori*. J Trop Pediatr. 2007;53(5):313–8. Epub 2007/05/31. 10.1093/tropej/fmm040 . 17535826

[17] Wazir S, Kumar L, Sethi S, Sharma M. Seroprevalence of Chlamydia pneumoniae in asthmatic children from Northern India. Indian Pediatr. 2007;44(2):133–6. Epub 2007/03/14. 17351305

[18] Nagy A, Keszei M, Kis Z, Budai I, Tolgyesi G, Ungvari I, et al. Chlamydophila pneumoniae infection status is dependent on the subtypes of asthma and allergy. Allergy Asthma Proc. 2007;28(1):58–63. Epub 2007/03/30. 10.2500/aap.2007.28.2957 . 17390759

[19] Kazar J, Kovacova E, Gasparovic J, Cervenka J, Furkova K, Hornova J, et al. Antibody response to chlamydiae in children with asthma and respiratory illness. Folia Microbiol (Praha). 2011. Epub 2011/04/20. 10.1007/s12223-011-0021-5 . 21503738

[20] Patel KK, Anderson EA, Salva PS, Webley WC. The prevalence and identity of Chlamydia-specific IgE in children with asthma and other chronic respiratory symptoms. Respir Res. 2012;13(1):32. Epub 2012/04/20. doi: 1465-9921-13-32 [pii] 10.1186/1465-9921-13-32 . 22512977 PMC3441249

[21] Tutanç M, Gürkan FM, Yel S, Güneş A, Konca Ç, Bilen G. Seropositivity for Chlamydia pneumoniae and Mycoplasma pneumoniae in asthmatic children. Journal of Clinical and Analytical Medicine. 2015;6(3):353–7. 10.4328/JCAM.2052

[22] Park SJ, Lee YC, Rhee YK, Lee HB. Seroprevalence of Mycoplasma pneumoniae and Chlamydia pneumoniae in stable asthma and chronic obstructive pulmonary disease. J Korean Med Sci. 2005;20(2):225–8. Epub 2005/04/16. doi: 200504225 [pii]. 10.3346/jkms.2005.20.2.225 15831991 PMC2808596

[23] Rodríguez JA, Munoz FJ, Bellido JLM, Garcia Rodríguez JA. Prevalence of anti-Chlamydophila pneumoniae antibodies in patients with intrinsic asthma. Rev Esp Quimioterap. 2005;18:146–8. PubMed Central PMCID: PMC16130036. 16130036

[24] Harju TH, Leinonen M, Nokso-Koivisto J, Korhonen T, Räty R, He Q, et al. Pathogenic bacteria and viruses in induced sputum or pharyngeal secretions of adults with stable asthma. Thorax. 2006;61(7):579–84. 10.1136/thx.2005.056291 PubMed Central PMCID: PMC16517571. 16517571 PMC2104650

[25] Paldanius M, Juvonen R, Leinonen M, Bloigu A, Silvennoinen-Kassinen S, Saikku P. Asthmatic persons are prone to the persistence of *Chlamydia pneumoniae* antibodies. Diagn Microbiol Infect Dis. 2007;59(2):117–22. Epub 2007/06/19. 10.1016/j.diagmicrobio.2007.04.004 . 17572038

[26] Kocabas A, Avsar M, Hanta I, Koksal F, Kuleci S. *Chlamydophila pneumoniae* infection in adult asthmatics patients. Journal of Allergy. 2008;45:39–43. 10.1080/02770900701815735 18259994

[27] Torshizi AA, Mohammad T, Attaran D, Karamadin MK, Ghazvini K. Role of *Chlamydia pneumoniae* infection in asthma in northeast of Iran. Iran J Allergy Asthma Immunol. 2008;7:45–6. doi: 07.01/ijaai.4546 . 18322313

[28] Dejsomritrutai W, Siritantikorn S, Nana A. Asthma, bronchial hyper-responsiveness and Chlamydophila (Chlamydia) pneumonia infection in adult Thai population. J Med Assoc Thai. 2009;92 Suppl 2:S30–7. Epub 2009/07/01. 10.2500/aap.2011.32.3431 . 19562983

[29] Specjalski K, Jassem E. *Chlamydophila pneumoniae*, *Mycoplasma pneumoniae* infections, and asthma control. Allergy Asthma Proc. 2011;32:9–17. PubMed Central PMCID: PMC21439159. 10.2500/aap.2011.32.3431 21439159

[30] Hahn DL, Schure A, Patel K, Childs T, Drizik E, Webley W. Chlamydia pneumoniae-specific IgE is prevalent in asthma and is associated with disease severity. PLoS ONE. 2012;7(4):e35945. 10.1371/journal.pone.0035945 22545149 PMC3335830

[31] Smith-Norowitz TA, Loeffler J, Huang Y, Klein E, Norowitz YM, Hammerschlag MR, et al. Chlamydia pneumoniae immunoglobulin E antibody levels in patients with asthma compared with non-asthma. Heliyon. 2020;6(2):e03512. Epub 2020/03/07. 10.1016/j.heliyon.2020.e03512 32140608 PMC7052057

[32] Pasternak R, Huhtala H, Karjalainen J. *Chlamydophila (Chlamydia) pneumoniae* serology and asthma in adults: a longitudinal analysis. J All Clin Immunol. 2005;116(5):1123–8. 10.1016/j.jaci.2005.08.030 16275386

[33] Schmidt SM, Muller CE, Wiersbitzky SK. Inverse association between Chlamydia pneumoniae respiratory tract infection and initiation of asthma or allergic rhinitis in children. Pediatr Allergy Immunol. 2005;16(2):137–44. Epub 2005/03/25. doi: PAI229 [pii] 10.1111/j.1399-3038.2005.00229.x . 15787871

[34] Zaitsu M. The development of asthma in wheezing infants with Chlamydia pneumoniae infection. J Asthma. 2007;44(7):565–8. Epub 2007/09/22. doi: 782170331 [pii] 10.1080/02770900701537115 17885860

[35] Zaitsu M. Does Chlamydia pneumoniae infection trigger to development of asthma in wheezy infants? J Asthma. 2009;46(9):967–8. Epub 2009/11/13. 10.3109/02770900903165582 [pii]. . 19905928

[36] Foschino Barbaro MP, Resta O, Aliani M, Guido P, Izzo C, Logroscino C, et al. Seroprevalence of chronic Chlamydia pneumoniae infection in patients affected by chronic stable asthma. Clin Microbiol Infect. 2002;8(6):358–62. Epub 2002/06/27. 10.1046/j.1469-0691.2002.00430.x . 12084104

[37] Gencay M, Rudiger JJ, Tamm M, Soler M, Perruchoud AP, Roth M. Increased frequency of Chlamydia pneumoniae antibodies in patients with asthma. Am J Respir Crit Care Med. 2001;163(5):1097–100. Epub 2001/04/24. 10.1164/ajrccm.163.5.2003162 . 11316642

[38] Hahn DL, Anttila T, Saikku P. Association of *Chlamydia pneumoniae* IgA antibodies with recently symptomatic asthma. Epidemiol Infect. 1996;117:513–7. 10.1017/s0950268800059197 8972677 PMC2271656

[39] Hahn DL, Peeling RW, Dillon E, McDonald R, Saikku P. Serologic markers for *Chlamydia pneumoniae* in asthma. Ann Allergy Asthma Immunol. 2000;84:227–33. 10.1016/S1081-1206(10)62760-3 10719781

[40] Larsen FO, Norn S, Mordhorst CH, Skov PS, Milman N, Clementsen P. *Chlamydia pneumoniae* and possible relationship to asthma. Serum immunoglobulins and histamine release in patients and controls. APMIS. 1998;106(10):928–34. PubMed Central PMCID: PMC9833693. 9833693

[41] Mills GD, Lindeman JA, Fawcett JP, Herbison GP, Sears M. Chlamydia pneumoniae serological status is not associated with asthma in children or young adults. Int J Epidemiol. 2000;29:280–4. 10.1093/ije/29.2.280 10817126

[42] Nagy A, Kozma GT, Keszei M, Treszi A, Falus A, Szalai C. The development of asthma in children infected with *Chlamydia pneumoniae* is dependent on the modifying effect of mannose-binding lectin. J Allergy Clin Immunol. 2003;112:729–34. 10.1016/s0091-6749(03)02010-4 14564351

[43] Routes JM, Nelson HS, Noda JA, Simon FT. Lack of correlation between *Chlamydia pneumoniae* antibody titers and adult-onset asthma. J Allergy Clin Immunol. 2000;105:391–2. 10.1016/s0091-6749(00)90093-9 PubMed Central PMCID: PMC10669864. 10669864

[44] Sirmatel F, Ustunsoy H, Sirmatel O, Akdemir I, Dikensoy O. The relationship between Chlamydia pneumoniae seropositivity and peripheral vascular diseases, acute myocardial infarction and late-onset asthma. Infection. 2003;31(5):367–8. Epub 2003/10/14. 10.1007/s15010-003-2130-9 . 14556067

[45] Tuuminen T, Edelstein I, Punin A, Kislova N, Stratchounski L. Use of quantitative and objective enzyme immunoassays to investigate the possible association between Chlamydia pneumoniae and Mycoplasma pneumoniae antibodies and asthma. Clin Microbiol Infect. 2004;10(4):345–8. Epub 2004/04/03. 10.1111/j.1198-743X.2004.00822.x [pii]. . 15059128

[46] von Hertzen L, Vasankari T, Liippo K, Wahlström E, Puolakkainen M. *Chlamydia pneumoniae* and severity of asthma. Scand J Infect Dis. 2002;34:22–7. 10.1080/00365540110077155 11874160

[47] Johnston SL, Blasi F, Black PN, Martin RJ, Farrell DJ, Nieman RB. The effect of telithromycin in acute exacerbations of asthma. N Engl J Med. 2006;354:1589–600. 10.1056/NEJMoa044080 16611950

[48] Johnston SL, Szigeti M, Cross M, Brightling C, Chaudhuri R, Harrison T, et al. Azithromycin for Acute Exacerbations of Asthma: The AZALEA Randomized Clinical Trial. JAMA Intern Med. 2016;176(11):1630–7. 10.1001/jamainternmed.2016.5664 . 27653939

[49] Hiles SA, McDonald VM, Guilhermino M, Brusselle GG, Gibson PG. Does maintenance azithromycin reduce asthma exacerbations? An individual participant data meta-analysis. Eur Respir J. 2019:10.1183/13993003.01381-2019 Epub 2019/09/14. . 31515407

[50] Steel HC, Theron AJ, Cockeran R, Anderson R, Feldman C. Pathogen- and host-directed anti-inflammatory activities of macrolide antibiotics. Mediators Inflamm. 2012;2012:584262. Epub 2012/07/11. 10.1155/2012/584262 22778497 PMC3388425

[51] Grayston JT, Campbell LA, Kuo C-C, Mordhorst CH, Saikku P, Thom DH, et al. A new respiratory tract pathogen: *Chlamydia pneumoniae* strain TWAR. J Infect Dis. 1990;161:618–25. 10.1093/infdis/161.4.618 2181028

[52] Burrows B, Martinez F, Halonen M, Barbee RA, Cline MG. Association of asthma with serum IgE levels and skin-test reactivity to allergens. New Engl J Med. 1989;320:271–7. 10.1056/NEJM198902023200502 2911321

[53] Schmidt SM, Müller CE, Bruns R, Wiersbitzky SKW. Bronchial *Chlamydia pneumoniae* infection, markers of allergic inflammation and lung function in children. Pediatr Allergy Immunol. 2001;12:257–65. 10.1034/j.1399-3038.2001.00042.x 11737672

[54] Wu L, Skinner SJM, Lambie N, Vuletic JC, Blasi F, Black PN. Immunohistochemical staining for *Chlamydia pneumoniae* is increased in lung tissue from subjects with chronic obstructive pulmonary disease. Am J Respir Crit Care Med. 2000;162:1148–51. 10.1164/ajrccm.162.3.9912134 10988144

[55] Webley WC, Salva PS, Andrzejewski C, Cirino F, West CA, Tilahun Y, et al. The bronchial lavage of pediatric patients with asthma contains infectious Chlamydia. American Journal of Respiratory & Critical Care Medicine. 2005;171(10):1083–8. 10.1164/rccm.200407-917OC . Language: English. Entry Date: 20050624. Revision Date: 20200624. Publication Type: Journal Article. 15735056

[56] Boman J, Söderberg S, Forsberg J, Birgander LS, Allard A, Persson K, et al. High prevalence of *Chlamydia pneumoniae* DNA in peripheral blood mononuclear cells in patients with cardiovascular disease and in middle-aged blood donors. Journal of Infectious Diseases. 1998;178:274–7. 10.1086/517452 9652454

[57] Parrott GL, Kinjo T, Fujita J. A Compendium for Mycoplasma pneumoniae. Front Microbiol. 2016;7:513. 10.3389/fmicb.2016.00513 27148202 PMC4828434

[58] Yeh JJ, Wang YC, Hsu WH, Kao CH. Incident asthma and Mycoplasma pneumoniae: A nationwide cohort study. J Allergy Clin Immunol. 2016;137(4):1017–23 e6. Epub 2015/11/21. 10.1016/j.jaci.2015.09.032 . 26586037

[59] Campbell LA, Hahn DL. *Chlamydia pneumoniae* Infections. In: Tan M, Hegemann JH, Sütterlin C, editors. Chlamydia Biology—From Genome to Disease. Norfolk, UK: Caister Academic Press; 2020. p. 31–58.

[60] Hahn DL. Role of Chlamydia pneumoniae as an inducer of asthma. In: Friedman H, Yamamoto Y, Bendinelli M, editors. *Chlamydia pneumoniae* Infection and Disease. Infectious Agents and Pathogenesis. New York: Kluwer Academic/Plenum Publishers; 2004. p. 239–62.

[61] Webley WC, Hahn DL. Infection-mediated asthma: etiology, mechanisms and treatment options, with focus on Chlamydia pneumoniae and macrolides. Respir Res. 2017;18(1):98. 10.1186/s12931-017-0584-z 28526018 PMC5437656

[62] Aaron SD, Vandemheen KL, FitzGerald JM, Ainslie M, Gupta S, Lemiere C, et al. Reevaluation of Diagnosis in Adults With Physician-Diagnosed Asthma. JAMA. 2017;317(3):269–79. 10.1001/jama.2016.19627 . 28114551

[63] Dowell SF, Peeling RW, Boman J, Carlone GM, Fields BS, Guarner J, et al. Standardizing Chlamydia pneumoniae assays: Recommendations from the Centers for Disease Control and Prevention (USA) and the Laboratory Centre for Disease Control (Canada). Clin Infect Dis. 2001;33:492–503. 10.1086/322632 11462186

[64] Herland K, Akselsen J-P, Skjønsberg OH, Bjermer L. How representative are clinical study patients with asthma or COPD for a larger "real life" population of patients with obstructive lung disease? Respir Med. 2005;99:11–9. 10.1016/j.rmed.2004.03.026 15672843

[65] Travers J, Marsh S, Williams M, Weatherall M, Caldwell B, Shirtcliffe P, et al. External validity of randomised controlled trials in asthma: to whom do the results of the trials apply? Thorax. 2007;62:219–23. 10.1136/thx.2006.066837 17105779 PMC2117157

[66] Hahn DL, Golubjatnikov R. Smoking is a potential confounder of the *Chlamydia pneumoniae*-coronary artery disease association. Arteriosclerosis & Thrombosis. 1992;12:945–7. 10.1161/01.atv.12.8.945 1637792

[67] Blasi F, Damato S, Cosentini R, Tarsia P, Raccanelli R, Centanni S, et al. Chlamydia pneumoniae and chronic bronchitis: association with severity and bacterial clearance following treatment. Thorax. 2002;57:672–6. 10.1136/thorax.57.8.672 12149525 PMC1746406

[68] Grayston JT, Aldous M, Easton A, Wang S-p, Kuo C-c, Campbell LA, et al. Evidence that *Chlamydia pneumoniae* causes pneumonia and bronchitis. J Infect Dis. 1993;168:1231–5. 10.1093/infdis/168.5.1231 8228356

[69] Hahn DL. A Cure for Asthma? What Your Doctor Isn’t Telling You, and Why. Durham, North Carolina: Peoples Pharmacy Press; 2013. 192 p.

