# Supplementary material for: Chlamydia pneumoniae and chronic asthma: Updated systematic review and meta-analysis of population attributable risk
Source: PLoS One. 2021 Apr 19;16(4):e0250034. doi: 10.1371/journal.pone.0250034 (PMC8055030; doi:10.1371/journal.pone.0250034)

**S3 Figure**. Funnel plot of 14 studies analyzed in Figure 3A. The two studies outside the triangle are Sirmatel 2003 and Specjalski 2011.


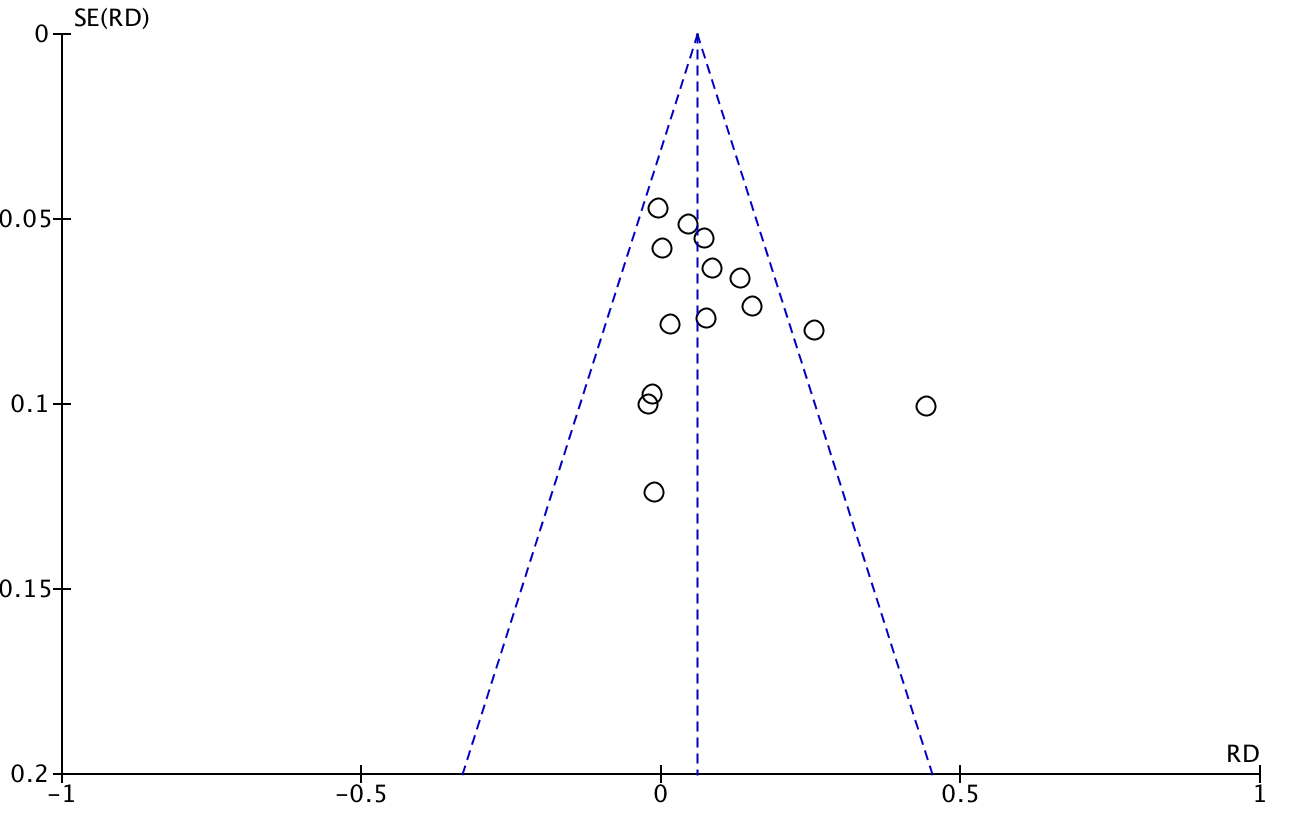

Supplement: S1 Fig — (DOCX) [file pone.0250034.s002.docx]
