# Supplementary material for: Chlamydia pneumoniae and chronic asthma: Updated systematic review and meta-analysis of population attributable risk
Source: PLoS One. 2021 Apr 19;16(4):e0250034. doi: 10.1371/journal.pone.0250034 (PMC8055030; doi:10.1371/journal.pone.0250034)

**S4 Figure**. Results of sensitivity analysis of severity subgroups (Figure 5) with Cp IgE seroreactivity results removed.


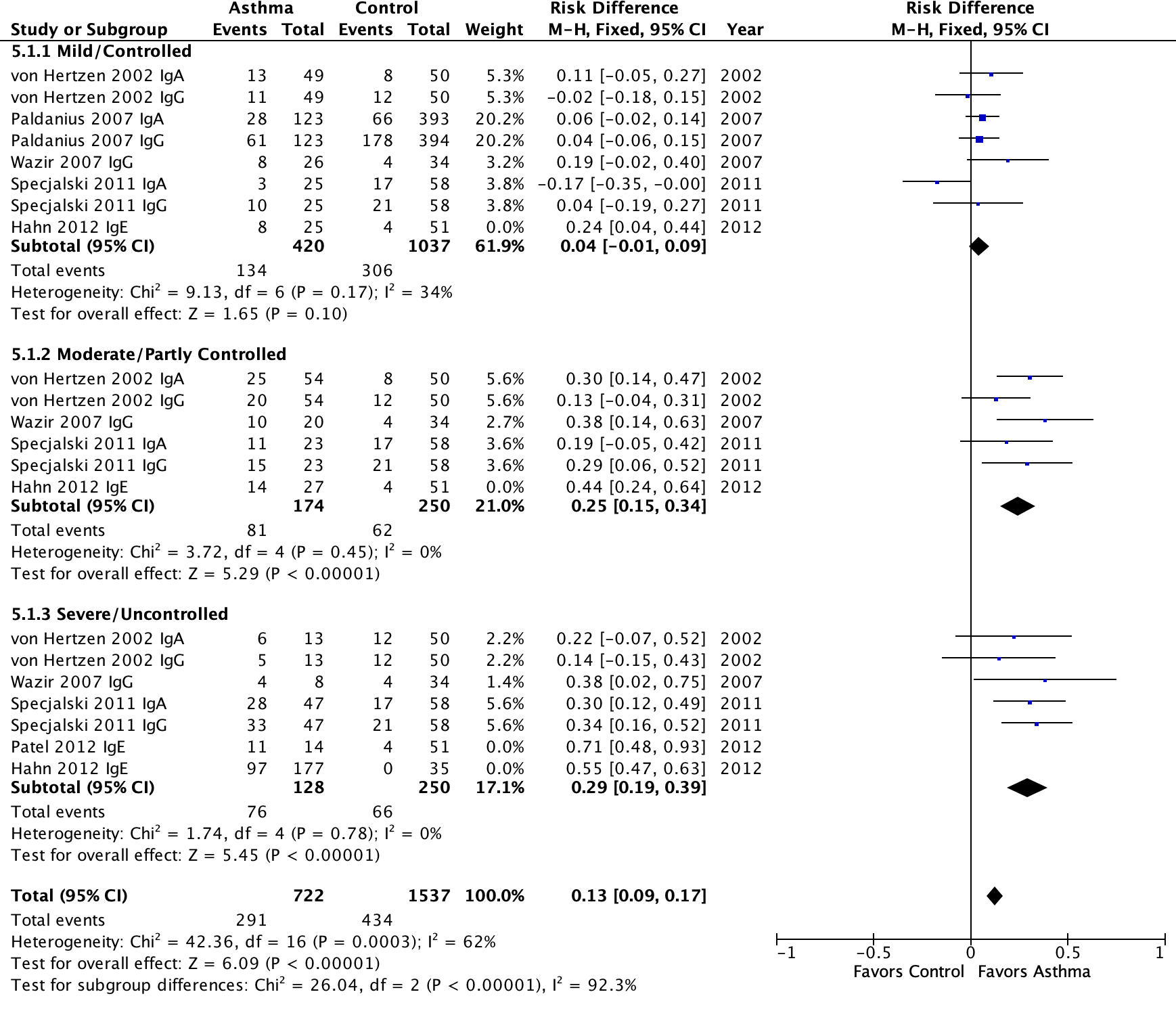

Supplement: S2 Fig — (DOCX) [file pone.0250034.s003.docx]
