# Supplementary material for: Chlamydia pneumoniae and chronic asthma: Updated systematic review and meta-analysis of population attributable risk
Source: PLoS One. 2021 Apr 19;16(4):e0250034. doi: 10.1371/journal.pone.0250034 (PMC8055030; doi:10.1371/journal.pone.0250034)
Supplement: S1 Appendix — (DOCX) [file pone.0250034.s004.docx]

**S1 Apendix. Search algorithms for updated systematic review**

**Pub Med**

(Asthma) AND (Chlamydia pneumoniae OR Chlamydophila pneumoniae)

Limits: 1/1/2000 – 10/17/2020

280 articles retrieved; 19 included in the updated systematic review

**Scopus**

((TITLE-ABS-KEY(Asthma) AND TITLE-ABS-KEY(Chlamydia pneumoniae OR Chlamydophila pneumoniae)) AND PUBYEAR > 1999 AND PUBYEAR < 2021)

320 articles retrieved; 1 additional articlle included in the systemtic review

**The Cochrane Library**

Asthma and (Chlamydia pneumoniae OR Chlamydophila pneumoniae)

Limits: January 2000 to October 2020

23 articles retrieved; no additioanl articles included in the systematic review

**CINAHL**

Asthma and (Chlamydia pneumoniae OR Chlamydophila pneumoniae)

Limits: January 2000 to October 2020

35 articles retrieved; no additional articles included in the systematic review
