# Supplementary material for: Chlamydia pneumoniae and chronic asthma: Updated systematic review and meta-analysis of population attributable risk
Source: PLoS One. 2021 Apr 19;16(4):e0250034. doi: 10.1371/journal.pone.0250034 (PMC8055030; doi:10.1371/journal.pone.0250034)
Supplement: S2 Appendix — (DOCX) [file pone.0250034.s005.docx]

**S2 Appendix. Quality assessment table for case-control studies included in the meta-analyses.**

Case selection Case diagnostic certainty Control selection Biomarker validation

| Hahn 1996 | Good | Good | Fair | Good |
| --- | --- | --- | --- | --- |
| Larsen 1998 | NR | Good | Fair | Good |
| Hahn 2000 | Good | Fair | Fair | Good |
| Mills 2000 | n/a | Good | Good | Good |
| Routes 2000 | NR | Good | Poor | Good |
| Gencay 2001 | Poor | Good | Fair | Good |
| Foschino Barbaro 2002 | Fair | NR | Poor | Good |
| von Hertzen 2002 | Poor | Good | Fair | Good |
| Nagy 2003 | n/a | Good | Fair | Fair |
| Sirmatel 2003 | Poor | NR | Fair | NR |
| Tuuminen 2004 | Fair | Good | Fair | Fair |
| Dal Molin 2005 | n/a | Fair | Good | Good |
| Rodriguez 2005 | NR | Fair | Fair | Fair |
| Annagur 2007 | n/a | Good | Poor | Fair |
| Nagy 2007 | n/a | Good | Fair | Fair |
| Paldanius 2007 | Good | Fair | Fair | Good |
| Wazir 2007 | n/a | Good | Fair | Fair |
| Kocabas 2008 | Fair | Good | Poor | Good |
| Dejsomritrutai 2009 | Good | Good | Good | Good |
| Kazar 2011 | n/a | Fair | Fair | Fair |
| Specjalski 2011 | Fair | Good | Poor | Fair |
| Hahn 2012 | Good | Fair | Fair | Good |
| Patel 2012 | n/a | Good | Fair | Good |
| Tutanç 2015 | n/a | Fair | Fair | Good |
| Smith-Norowitz 2020 | NR | Fair | Fair | Fair |

**Case selection:** Good (smoking and lung co-morbidities included); Fair (smoking included); Poor (neither included)

{n/a for pediatric asthma}

**Case diagnostic certainty:** Good (physician-diagnosed/self/parent-reported asthma supported by systematic objective testing); Fair (physician-diagnosed/self/parental-reported asthma without systematic objective testing)

**Control selection:** Good (random population sample); Fair (convenience sample of healthy controls); Poor (restricted sample, e.g., family/health care workers or inappropriate matching)

**Biomarker validation:** Good (MIF testing); Fair (ELISA/other)

**n/a:** not applicable

**NR:** not reported
