# Supplementary material for: Chlamydia pneumoniae and chronic asthma: Updated systematic review and meta-analysis of population attributable risk
Source: PLoS One. 2021 Apr 19;16(4):e0250034. doi: 10.1371/journal.pone.0250034 (PMC8055030; doi:10.1371/journal.pone.0250034)
Supplement: S1 File — (DOCX) [file pone.0250034.s006.docx]

**Research in context**

**Evidence before this study**

Macrolides are now a recommended treatment option for severe asthma based on the positive results of two randomized, controlled trials, however underlying mechanism(s) of action is/are unknown. *Chlamydia pneumoniae* (Cp) chronic infection is prevalent in human populations worldwide and it has been hypothesized that an aberrant host response to this chronic infection contributes to severe asthma pathogenesis. Previous reviews of Cp infection and asthma have concluded that there are positive associations of Cp and asthma, and that further research is required to investigate therapeutic implications of this association.

**Added value of this study**

This updated systematic review confirms that Cp biomarkers continue to be associated with asthma. There are now sufficient data to perform a meta-analysis of Cp biomarker population attributable risks (i.e., the percent of asthma that could possibly be prevented or improved if the risk factor were to be removed from a population). Results showed that the population attributable risk for Cp-specific IgE was 47% (95% confidence interval 40% to 55%) and that Cp biomarkers were strongly associated with increased asthma severity.

**Implication of all the available evidence**

The evidence suggests that chronic Cp infection is associated with asthma severity and that an IgE-mediated host immune response to this unrecognized “allergen” is a plausible mechanism in asthma pathogenesis in the subset of infected patients. Future asthma-macrolide trials should include Cp in their biomarker arrays. Clinicians should keep in mind the possibility that they are treating an infection as they prescribe macrolides for asthma, and consider following a recommendation of the British Thoracic Society that “ongoing treatment should be guided by clinical response based on specific outcome measures, including exacerbation frequency, symptoms and quality of life assessed at baseline.”
