# Supplementary material for: Chlamydia pneumoniae and chronic asthma: Updated systematic review and meta-analysis of population attributable risk
Source: PLoS One. 2021 Apr 19;16(4):e0250034. doi: 10.1371/journal.pone.0250034 (PMC8055030; doi:10.1371/journal.pone.0250034)
Supplement: S1 Dataset — (PDF) [file pone.0250034.s007.pdf]

Case-Control and Cohort studies reporting biomarker prevalence data allowing calculatin of population attributable risk (PAR)

**AUTHOR**<br>DOI OR PMID	**Biomarker**<br>Peripheral blood unless otherwise	**Criteria for +**<br>other than present/abse	**#Cases**	**#Cases**	**%Cases+**	**#Controls**	**#Controls+**	**%Controls+**	**AR (%)**	**Comment**
**ADULT ASTHMA**										
**CASE-CONTROL (OR NESTED CASE/CONTROL)**										
Hahn & Golubjatnikov (1994)<br>**8195733**	Poylvalent (IgM, IgG, IgA) MIF	≥1:16	12	12	100.0%	89	47	52.8%	**47.2%**	Persistent chronic asthma, outpatient
Cook et al. (1998)<br>10.1136/thx.53.4.254	IgM, IgG, IgA MIF	IgG 64-256 or IgA≥8	46	16	34.8%	1518	193	12.7%	**22.1%**	Severe chronic asthma, outpatient
Hahn et al. (2000)	IgG MIF	≥1:16	68	63	92.6%	44	37	84.1%	**8.6%**	AAWI: asthma associated with infection at onset of first reported symptoms
Hahn et al. (2000)<br>10.1016/S1081-1206(10)62760-3	IgA MIF	≥1:16	68	47	69.1%	44	19	43.2%	**25.9%**	AAWI: asthma associated with infection at onset of first reported symptoms

Gencay et al. (2001)	IgG MIF	≥1:32	33	21	63.6%	33	19	57.6%	**6.1%**	Asthma severity not specified, All cases and controls were non-smokers.
Gencay et al. (2001)	IgA MIF	≥1:20	33	17	51.5%	33	9	27.3%	**24.2%**	Asthma severity not specified, All cases and controls were non-smokers.
Gencay et al. (2001)	IgG MIF	IgG≥1:512	33	6	18.2%	33	1	3.0%	**15.2%**	Asthma severity not specified, All cases and controls were non-smokers.
Gencay et al. (2001)	IgG & IgA MIF	IgG≥1:512 and IgA≥1:20	33	6	18.2%	33	1	3.0%	**15.2%**	Asthma severity not specified, All cases and controls were non-smokers.
10.1164/ajrccm.163.5.2003162										
Foschino Barbaro et al. (2002)	IgG by MIF	IgG≥1:16	197	60	30.5%	185	57	30.8%	**-0.4%**	Only 17 (8.6%) of 197 asthma cases were severe asthma; controls were hospital staff, not general community.
10.1046/j.1469-0691.2002.00430.x.										
von Hertzen et al.	IgG MIF	≥1:256	49	11	22.4%	50	12	24.0%	**-1.6%**	Mild asthma
von Hertzen et al.	IgG MIF	≥1:256	54	20	37.0%	50	12	24.0%	**13.0%**	Moderate asthma
von Hertzen et al.	IgG MIF	≥1:256	13	5	38.5%	50	12	24.0%	**14.5%**	Severe asthma
von Hertzen et al.	IgG MIF	≥1:256	116	36	31.0%	150	36	24.0%	**7.0%**	TOTAL
von Hertzen et al.	IgA MIF	≥320	49	13	26.5%	50	8	16.0%	**10.5%**	Mild asthma
von Hertzen et al.	IgA MIF	≥320	54	25	46.3%	50	8	16.0%	**30.3%**	Moderate asthma
von Hertzen et al.	IgA MIF	≥320	13	6	46.2%	50	8	16.0%	**30.2%**	Severe asthma
von Hertzen et al.	IgA MIF	≥320	116	44	37.9%	150	24	16.0%	**21.9%**	TOTAL
10.1080/00365540110077155										

Biscione et al. (2004)	nested RT-PCR on MOMP mRNA	any + nasal secretion sampled q2w for 3 months	74	16	21.6%	74	7	9.5%	**12.2%**	Asthma was atopic, mild-moderate; non-atopic spouse was the control; smokers were excluded.
10.1183/09031936.04.00049004										
Martin et al. (2001)	IgM, IgG by MIF	IgG≥1:512 or IgM≥1:16 or	54	18	33.3%	11	1	9.1%	**24.2%**	Poor agreement between serology and PCR
10.1067/mai.2001.113563										
Tuuminen et al.	IgG by EIA	>45	150	78	52.0%	150	78	52.0%	**0.0%**	Asthma severity not specified.
	IgA by EIA	>12	150	56	37.3%	150	46	30.7%	**6.7%**	Asthma severity not specified.
10.1111/j.1198-743X.2004.00822.x										
Hahn et al. (1996)	IgG by MIF	≥1:16	25	23	92.0%	45	38	84.4%	**7.6%**	Asthma onset within 2 years, severity not specified.
	IgA by MIF	≥1:10	25	18	72.0%	45	20	44.4%	**27.6%**	Asthma onset within 2 years, severity not specified.
10.1017/s0950268800059197										
Larsen et al. (1998)	IgG by MIF	≥1:16	22	5	22.7%	25	6	24.0%	**-1.3%**	The majority of cases were atopic, severity not specified; all controls were non-atopic. Lower than expected populstion prevalence.
**9833693**										

Routes et al. (2000)	IgG by MIF	≥1:64	46	29	63.0%	46	30	65.2%	**-2.2%**	Asthma onset after age 40; age- and sex-matched control drawn from hospitalized patients and hospital workers.
10.1016/s0091-6749(00)90093-9										
Sirmatel et al. (2003)	IgG by MIF	≥1:32	25	15	60.0%	28	14	50.0%	**10.0%**	Mild late-onset non-atopic asthmatic non-smokers.
	IgG by MIF	≥1:64	25	11	44.0%	28	0	0.0%	**44.0%**	Mild late-onset non-atopic asthmatic non-smokers.
	IgA by MIF	≥1:16	25	4	16.0%	28	0	0.0%	**16.0%**	Mild late-onset non-atopic asthmatic non-smokers.
10.1007/s15010-003-2130-9										
Park et al. (2005)	IgM, IgG by MIF	IgG>1;512 or IgM>1:20	36	3	8.3%	45	1	2.2%	**6.1%**	No biomarker data for lesser amounts of seroreactivity provided.
200504225 [pii]										
Rodriguez et al. (2005)	IgG by ELISA against Cp LPS	OD>1.1	55	13	23.6%	87	9	10.3%	**13.3%**	Asthma severity not specified. Intrinsic asthma.
**16130036**										
Harju et al. (2006)	Cp PCR of nasal swabs and/or		53	10	18.9%	30	9	30.0%	**-11.1%**	Mild asthma
Harju et al. (2006)	Cp PCR of nasal swabs and/or		50	8	16.0%	30	9	30.0%	**-14.0%**	Moderate asthma
10.1136/thx.2005.056291										

Paldanius et al. (2007)	IgG by MIF	IgG≥1:32	123	61	49.6%	394	178	45.2%	**4.4%**	Mild asthma (miltary recruits)
Paldanius et al. (2007)	IgA by MIF	IgA≥1:10	123	28	22.8%	393	66	16.8%	**6.0%**	Mld asthma (military recruits)
10.1016/j.diagmicrobio.2007.04.004										
Kocabas et al. (2008)	Cp IgG by MIF	IgG≥1:16	84	53	63.1%	34	22	64.7%	**-1.6%**	32 mild intermittent, 38 mild persistent, 13 moderate, 1 severe
	PCR of throat wash		84	24	28.6%	34	4	11.8%	**16.8%**	32 mild intermittent, 38 mild persistent, 13 moderate, 1 severe
10.1080/02770900701815735										
Torshizi et al. (2008)	Culture of NP		20	7	35.0%	20	1	5.0%	**30.0%**	Asthma severity not specified.
07.01/ijaai.4546										
Dejsomritrutai et al. (2009)	IgG by MIF	IgG≥1:32	52	33	63.5%	137	85	62.0%	**1.4%**	Nested case-control study from a population-based cohort. Asthma severity not specified. Did not require currently symptomatic asthma.
Dejsomritrutai et al. (2009)	IgA by MIF	IgA≥1:32	52	15	28.8%	137	30	21.9%	**6.9%**	Nested case-control study from a population-based cohort. Asthma severity not specified. Did not require currently symptomatic asthma.
**19562983**										
Specjalski et al. (2011)	IgG by ELISA	Seropositivity per Savyon	95	58	61.1%	58	21	36.2%	**24.8%**	25 (26.3%) controlled, 23 (24.2%) partly controlled, 47 (49.5%) uncontrolled.

	IgG by ELISA	Seropositivity	25	10	40.0%	58	21	36.2%	**3.8%**	controlled
	IgG by ELISA	Seropositivity	23	15	65.2%	58	21	36.2%	**29.0%**	partly controlled
	IgG by ELISA	Seropositivity	47	33	70.2%	58	21	36.2%	**34.0%**	uncontrolled
Specjalski et al. (2011)	IgA by ELISA	Seropositivity per Savyon	95	42	44.2%	58	17	29.3%	**14.9%**	25 (26.3%) controlled, 23 (24.2%) partly controlled, 47 (49.5%) uncontrolled.
	IgA by ELISA	Seropositivity	25	3	12.0%	58	17	29.3%	**-17.3%**	controlled
	IgA by ELISA	Seropositivity	23	11	47.8%	58	17	29.3%	**18.5%**	partly controlled
	IgA by ELISA	Seropositivity	47	28	59.6%	58	17	29.3%	**30.3%**	uncontrolled
10.2500/aap.2011.32.3431										
Hahn et al. (2012)	Cp-specific IgE by immunoblotting		25	8	32.0%	51	4	7.8%	**24.2%**	Mild intermittent and persistent asthma
Hahn et al. (2012)	Cp-specific IgE by immunoblotting		27	14	51.9%	51	4	7.8%	**44.0%**	Moderate persistent asthma
Hahn et al. (2012)	Cp-specific IgE by immunoblotting		14	11	78.6%	51	4	7.8%	**70.7%**	Severe persistent asthma
Hahn et al. (2012)	Cp-specific IgE by immunoblotting		66	33	50.0%	153	12	7.8%	**42.2%**	TOTAL
10.1371/journal.pone.0035945										
Smith-Norowitz et al. (2020)	Cp-IgE by EIA	OD≥0.464	22	21	95.5%	22	10	45.5%	**50.0%**	Asthma severity not specified. All subjects were Cp IgG seropositive.
10.1016/j.heliyon.2020.e03512										

COHORT

Pasternak et al. (2005)	Cp IgG by MIF	IgG≥1:32	83	53	63.9%	162	111	68.5%	**-4.7%**	Asthma severity not specified. NOT ELIGIBLE (does not meet case definition)
Pasternak et al.	Cp IgA by Mif	IgA≥1:8	83	39	47.0%	162	101	62.3%	**-15.4%**	Asthma severity not specified.
10.1016/j.jaci.2005.08.030										
**PEDIATRIC ASTHMA**	Peripheral blood unless otherwise									
**CASE-CONTROL**										
Emre 1995	IgE by immunoblotting		14	12	85.7%	31	5	16.1%	**69.6%**	CASES=Culture positive wheezing asthmatic children/TOTAL CONTROLS
Emre 1995	IgE by immunoblotting		14	12	85.7%	11	2	18.2%	**67.5%**	CONTROL=Culture negative asthmatic children
Emre 1995	IgE by immunoblotting		14	12	85.7%	11	1	9.1%	**76.6%**	CONTROL=Culture positive pneumonia patients
Emre 1995	IgE by immunoblotting		14	12	85.7%	9	2	22.2%	**63.5%**	CONTROL=Culture negative asymptomatic children
Emre 1995	IgE by immunoblotting		25	14	56.0%	20	3	15.0%	**41.0%**	CASES=All asthma children/TOTAL CONTROLS. NOT ELIGIBLE (AEX)
Emre 1995	IgE by immunoblotting		25	14	56.0%	11	1	9.1%	**46.9%**	CONTROL=Culture positive pneumonia patients
Emre 1995	IgE by immunoblotting		25	14	56.0%	9	2	22.2%	**33.8%**	CONTROL=Culture negative asymptomatic children
10.1093/infdis/172.1.265										
Mills et al. (2000)	IgG MIF	≥1:16	96	52	54.2%	102	64	62.7%	**-8.6%**	Self-reported ever-asthma, mostly mild
10.1093/ije/29.2.280										

Nagy et al. (2003)	IgG by ELISA	≥1.1	135	59	43.7%	173	82	47.4%	**-3.7%**	Asthma severity not reported
	IgA by ELISA	≥1.1	137	43	31.4%	174	56	32.2%	**-0.8%**	Asthma severity not reported
10.1016/s0091-6749(03)02010-4										
Webley et al. (2005)	Blood culture for Cp		42	17	40.5%	70	8	11.4%	**29.0%**	Severe asthma patients undergoing bronchoscopy
10.1164/rccm.200407-917OC										
Kopriva et al. (2005)	IgM, IgG, IgA by ELISA	IgM and IgG or IgG and IgA, cutoff	66	29	43.9%	241	38	15.8%	**28.2%**	Cases were atopic and had mild asthma. Controls were non-atopic.
10.5507/bp.2005.044										
Teig et al. (2005)	PCR on nasal brushings and/or		26	4	15.4%	42	0	0.0%	**15.4%**	Asthma severity not reported.
10.1136/thx.2005.041004										
Dal Molin et al. (2005)	IgG by MIF	Criterion for seropositivity not presented	47	8	17.0%	602	180	29.9%	**-12.9%**	Asthma was by self-report. Control group did not exclude subjects with rrespiratory ailments. Asthma severity not reported.
Dal Molin et al. (2005)	IgA by MIF	Criterion for seropositivity not presented	47	5	10.6%	602	123	20.4%	**-9.8%**	Asthma was by self-report. Control group did not exclude subjects with rrespiratory ailments. Asthma severity not reported.
10.1136/jcp.2004.024380										

Annagur et al. (2007)	IgG by ELISA	Criterion for seropositivity not presented	42	17	40.5%	36	8	22.2%	**18.3%**	IgM was not detected in this population of chronic stable asthma but was detected in some asthma patients in exacerbation in a different study group.
10.1136/jcp.2004.024380										
Wazir et al. (2007)	IgG by ELISA	Criterion for seropositivity	26	8	30.8%	34	4	11.8%	**19.0%**	Mild asthma
Wazir et al. (2007)	IgG by ELISA	Criterion for seropositivity	20	10	50.0%	34	4	11.8%	**38.2%**	Moderate asthma
Wazir et al. (2007)	IgG by ELISA	Criterion for seropositivity	8	4	50.0%	34	4	11.8%	**38.2%**	Severe asthma
Wazir et al. (2007)	IgG by ELISA	Criterion for seropositivity	54	22	40.7%	102	12	11.8%	**29.0%**	TOTAL
**17351305**										
Nagy et al. (2007)	IgG by ELISA	Seropositivity per Savyon,	141	61	43.3%	187	90	48.1%	**-4.9%**	Severe asthma not included=mild
Nagy et al. (2007)	IgA by ELISA	Seropositivity per Savyon,	141	45	31.9%	187	61	32.6%	**-0.7%**	Severe asthma not included=mild
10.2500/aap.2007.28.2957										
Kazar et al. (2011)	IgG by ELISA	OD≥1.1	97	19	19.6%	129	26	20.2%	**-0.6%**	46 mild intermittent, 40 mild persistent, 11 moderate persistent asthma
Kazar et al. (2011)	IgA by ELISA	OD≥1.1	97	14	14.4%	129	18	14.0%	**0.5%**	46 mild intermittent, 40 mild persistent, 11 moderate persistent asthma

10.1007/s12223-011-0021-5										
Patel et al. (2012)	Cp-specific IgE by immunoblotting of serum		177	97	54.8%	35	0	0.0%	**54.8%**	Cases had severe inflammatory lung diseases, unresponsive to corticosteroids; 143 had severe refractory asthma. Results for asthma subgroup noy reported separately.
10.1186/1465-9921-13-32										
Tutanç et al. (2015)<br>10.4328/JCAM.2052	Cp IgG by indirect immunofluoresce nce	Titre ≥1/100	66	18	27.3%	46	6	13.0%	**14.2%**	Positive association with more frequent attacks, but overall severity not specified
